# Multi-Omics Analyses Reveal the Regulatory Network and the Function of ZmUGTs in Maize Defense Response

**DOI:** 10.3389/fpls.2021.738261

**Published:** 2021-09-24

**Authors:** Chunxia Ge, Yi-Ge Wang, Shouping Lu, Xiang Yu Zhao, Bing-Kai Hou, Peter J. Balint-Kurti, Guan-Feng Wang

**Affiliations:** ^1^The Key Laboratory of Plant Development and Environmental Adaptation Biology, Ministry of Education, School of Life Sciences, Shandong University, Qingdao, China; ^2^School of Public Health and Management, Binzhou Medical University, Yantai, China; ^3^Maize Research Institute, Shandong Academy of Agricultural Sciences, Jinan, China; ^4^State Key Laboratory of Crop Biology, Shandong Agricultural University, Tai'an, China; ^5^Department of Entomology and Plant Pathology, North Carolina State University, Raleigh, NC, United States; ^6^US Department of Agriculture-Agricultural Research Service, Plant Science Research Unit, Raleigh, NC, United States

**Keywords:** disease resistance, ETI, hypersensitive response, maize, NLR, salicylic acid, UGT

## Abstract

Maize is one of the major crops in the world; however, diseases caused by various pathogens seriously affect its yield and quality. The maize *Rp1-D21* mutant (mt) caused by the intragenic recombination between two nucleotide-binding, leucine-rich repeat (NLR) proteins, exhibits autoactive hypersensitive response (HR). In this study, we integrated transcriptomic and metabolomic analyses to identify differentially expressed genes (DEGs) and differentially accumulated metabolites (DAMs) in *Rp1-D21* mt compared to the wild type (WT). Genes involved in pathogen-associated molecular pattern (PAMP)-triggered immunity (PTI) and effector-triggered immunity (ETI) were enriched among the DEGs. The salicylic acid (SA) pathway and the phenylpropanoid biosynthesis pathway were induced at both the transcriptional and metabolic levels. The DAMs identified included lipids, flavones, and phenolic acids, including 2,5-DHBA *O*-hexoside, the production of which is catalyzed by uridinediphosphate (UDP)-dependent glycosyltransferase (UGT). Four maize *UGTs* (*ZmUGTs)* homologous genes were among the DEGs. Functional analysis by transient co-expression in *Nicotiana benthamiana* showed that ZmUGT9250 and ZmUGT5174, but not ZmUGT9256 and ZmUGT8707, partially suppressed the HR triggered by Rp1-D21 or its N-terminal coiled-coil signaling domain (CC_D21_). None of the four ZmUGTs interacted physically with CC_D21_ in yeast two-hybrid or co-immunoprecipitation assays. We discuss the possibility that ZmUGTs might be involved in defense response by regulating SA homeostasis.

## Introduction

To defend against pathogenic microorganisms, plants have evolved a multilayered and sophisticated immune system including pathogen-associated molecular pattern (PAMP)-triggered immunity (PTI) and effector-triggered immunity (ETI; McHale et al., 2006; Cui et al., 2015). The PTI is activated by the recognition of PAMPs *via* the pattern recognition receptors (PRRs) localized at the surface of plant cells, while ETI is triggered when the intracellular receptors termed nucleotide-binding leucine-rich-repeat (NLR) proteins recognize the specific effector proteins secreted from pathogens. Two recent studies (Ngou et al., 2021; Yuan et al., 2021) suggest that PTI and ETI responses are closely connected with ETI potentiating the PTI response. PRR- and NLR-mediated downstream signaling pathways result in some similar immune outputs, such as increased expression of pathogenesis-related (*PR*) genes and the burst of reactive oxygen species (ROS). A nicotinamide adenine dinucleotide phosphate (NADPH) oxidase called respiratory burst oxidase homolog D (RBOHD) is an indispensable immune component connecting PRR and NLR immune receptors, and the phosphorylation of RBOHD mediates ROS generation and activates disease resistance (Ngou et al., 2021). A distinct feature of NLR-triggered immunity is often accompanied by the hypersensitive response (HR), a form of localized programmed cell death at the pathogen infection sites (Bent and Mackey, 2007; Kourelis and van der Hoorn, 2018; Balint-Kurti, 2019).

Salicylic acid (SA) is a pivotal phytohormone mediating both local and systemic defense responses against biotrophic and semi-biotrophic pathogens (Vlot et al., 2009; Dempsey et al., 2011). The activation of SA biosynthesis, metabolism, and signaling pathways play critical roles for both PTI- and ETI-mediated defense responses (Vlot et al., 2009; Ding et al., 2018). The SA is synthesized by the isochorismate synthase (ICS) and the Phe ammonia-lyase (PAL) pathways which play the major and minor roles in SA biosynthesis, respectively (Dempsey et al., 2011). In Arabidopsis, when the gene *EDS16* in the ICS pathway was mutated, the total SA level was reduced to <10% of the wild-type level after *Erysiphe orontii* infection (Dewdney et al., 2000). Once synthesized, the SA is subject to a number of modifications including glycosylation, hydroxylation, and methylation. Hydroxylated products of SA, including 2,3-dihydroxybenzoic acid (2,3-DHBA) and 2,5-dihydroxybenzoic acid (2,5-DHBA), are the major metabolic forms of SA. The DHBA is cytotoxic and it is generally found in its less toxic glycosylated form in plants (Bartsch et al., 2010). Glycosylation products of DHBA may activate plant defense. DHBA glycoside compounds are increased in Arabidopsis after infection by *Pseudomonas syringae* pv. *tomato* (*Pst*) strain DC3000 or *Hyaloperonospora arabidopsidis* (Bartsch et al., 2010). When overexpressed, the Arabidopsis uridine diphosphate (UDP)-dependent glycosyltransferase UGT76D1, which catalyzes the formation of DHBA glycosides, leads to the induction of HR, a burst of ROS, the increased expression of *PR* genes and enhanced resistance to *Pst* DC3000 (Huang et al., 2018). The UGT76D1 plays important roles in plant immunity by modulating SA homeostasis by glycosylations of DHBA (Huang et al., 2018).

Nucleotide-binding leucine-rich-repeat disease resistance proteins can be divided into two major types depending on their N-terminal domains; the coiled-coil type (CNL) and the Toll/interleukin-1 receptor type (TNL; Monteiro and Nishimura, 2018; Sun et al., 2020). The maize *Rp1* locus on the short arm of chromosome 10 carries multiple tandemly repeated CNL genes (Hulbert, 1997). One of these genes, *Rp1-D*, confers resistance to maize common rust caused by the fungus, *Puccini sorghi* (Hulbert, 1997). The chimeric gene *Rp1-D21* was derived from intragenic recombination between two paralogs, *Rp1-D* and *Rp1-dp2* (Sudupak et al., 1993; Sun et al., 2001; Smith et al., 2010). The *Rp1-D21* confers a spontaneous HR phenotype in the absence of pathogen infection (Sun et al., 2001; Smith et al., 2010; Wang et al., 2015a). The severity of this HR is affected by light, temperature, developmental stage, and genetic background (Chintamanani et al., 2010; Negeri et al., 2013). The *Rp1-D21*-induced HR is entirely suppressed at 30°C and can be activated by reducing the temperature to 22°C (Negeri et al., 2013). The *Rp1-D21* had been used as a tool to identify quantitative trait loci (QTL), genes, and pathways associated with modulation of the severity of HR in maize (Chintamanani et al., 2010; Olukolu et al., 2014). Two key enzymes in lignin biosynthesis pathway, caffeoyl-CoA O-methyltransferase (CCoAOMT) and hydroxycinnamoyl transferase (HCT), have been shown to suppress Rp1-D21-induced HR through physical interaction (Wang et al., 2015b; Wang and Balint-Kurti, 2016). The gene encoding CCoAOMT has also been shown to increase resistance to both southern leaf blight and gray leaf spot in maize (Yang et al., 2017).

In recent years, transcriptome and metabolome analyses have provided a powerful comprehensive approach to assess the relationship of genotype, phenotype, and metabolite changes in plants challenged by abiotic and biotic stresses (Etalo et al., 2013; Mo et al., 2019; Ye et al., 2019; Hong et al., 2020). For example, transcriptional profiling combined with targeted metabolite quantification found that the levels of many genes and several metabolites in phenylpropanoid and shikimate pathways are significantly changed by the expression of WtsE, an effector secreted from *Pantoea stewartii* ssp. *Stewartii* (*Pnss*), which can cause Stewart's wilt and leaf blight in maize (Asselin et al., 2015).

Here, using a similar multi-omics approach, we investigated the gene regulatory network modulating Rp1-D21-mediated HR. We identified a number of different pathways associated with resistance response mediated by *Rp1-D21*, in particular, the SA biosynthesis and metabolism pathway. Four DHBA glucosyl-transferase homologs of *ZmUGTs*, were highly induced, two of which we show may have important roles in modulating Rp1-D21-mediated HR.

## Results

### Transcriptome Sequencing and Quality Assessment

Transcriptional analysis of *Rp1-D21*-induced hypersensitive response (HR) was undertaken in two different temperature regimes (Supplementary Figure 1). In the first treatment, here called the temperature shift treatment, plants were grown at 30°C, and then were transferred immediately to 22°C to induce a synchronous systematic HR. For the second temperature regime, the constant temperature treatment, the plants were grown at a constant 22°C. Comparisons of two different pairs of near isogenic F1 hybrids were used for these experiments: B73 × H95-*Rp1-D21* with B73 × H 95 and Mo17 × H95-*Rp1-D21* with Mo17 × H95.

The *Rp1-D21* HR phenotype in Mo17 × H95-*Rp1-D21* is more severe than in B73 × H95-*Rp1-D21* (Chintamanani et al., 2010). In the temperature shift experiment, *Rp1-D21*-carrying plants displayed HR at 3 days after temperature shift in the Mo17 × H95-*Rp1-D21*, and at 5 days in the B73 × H95-*Rp1-D21* background. For this experiment, samples from near-isogenic wild type (WT) and *Rp1-D21* mutant (mt) plants in each genetic background were collected at 3, 6, 24, and 48 h post the temperature shift (hpts). The HR was not observed in either background at these time points. Therefore, in order to confirm that the defense response had indeed been activated by the temperature shift, we used semi-quantitative reverse transcription polymerase chain reaction (RT-PCR) to monitor the transcript levels of *PR1* and *PR5*, the two plant defense response marker genes. The *PR1* levels were noticeably induced in mutant plants at 6 hpts in Mo17 × H95 background and at 24 hpts in the B73× H95 background, while the *PR5* levels increased in mutant plants at 24 hpts and 48 hpts in B73 × H95 and Mo17 × H95, respectively (Supplementary Figure 2). Thus, we chose samples collected at 6 and 48 hpts for RNA sequencing (RNA-seq) experiments to investigate the early response genes involved in *Rp1-D21*-induced HR. These samples are referred to below as B73 × H95-WT-6 hpts, B73 × H95-mt-6 hpts, B73 × H95-WT-48 hpts, B73 × H95-mt-48 hpts, Mo17 × H95-WT-6 hpts, Mo17 × H95-mt-6 hpts, Mo17 × H95-WT-48 hpts, and Mo17 × H95-mt-48 hpts. To investigate whether the expression of *Rp1-D21* was induced after temperature shift which induced HR, we performed semi-quantitative RT-PCR analysis and found that the transcript levels of *Rp1-D21* were increasingly induced from 3 to 48 hpts in both genetic backgrounds (Supplementary Figure 2).

We used the constant temperature experiment to explore the late response genes involved in *Rp1-D21*-induced HR. In this case, we sampled plants from near isogenic pairs of each genetic background and the sample names were B73 × H95-WT-22°C, B73 × H95-mt-22°C, Mo17 × H95-WT-22°C, and Mo17 × H95-mt-22°C.

Read numbers for each sample ranged from 21.48 to 44.06 million with an average of 32.36 million. In each case, the read number of WT and mt from the same background at the same time point were quite similar (Supplementary Table 1). Hierarchical indexing for spliced alignment of transcripts 2 (HISAT2) was used to map the reads against the B73 reference genome (B73_v4, maizegdb.org). Approximately 87.57–92.92% of the reads were mapped to the reference genome, of which 84.79% were uniquely mapped (Supplementary Table 1). Fragments per kilobase of exon per million mapped reads (FPKM) values were calculated to evaluate the gene expression levels and the reproducibility of biological replicates. Raw counts expression data showed an average Pearson's correlation coefficient of 0.87 ranging from 0.65 to 0.96, indicating high correlation between the two biological replicates in each case (Supplementary Figure 3A). A multidimensional scaling (MDS) plot was generated to assess the sequencing quality (Supplementary Figure 3B). The transcript levels of all the expressed genes were clearly divided into different groups at constant 22°C for WT and *Rp1-D21* mt from B73× H95 and Mo17 × H95 backgrounds, but it was difficult to distinguish for samples at 6 hpts, which might be due to the low number of differentially expressed genes (DEGs) detected at this timepoint.

### Identification of DEGs Responsive to *Rp1-D21*-Induced Hypersensitive Response

**Figure 1:**
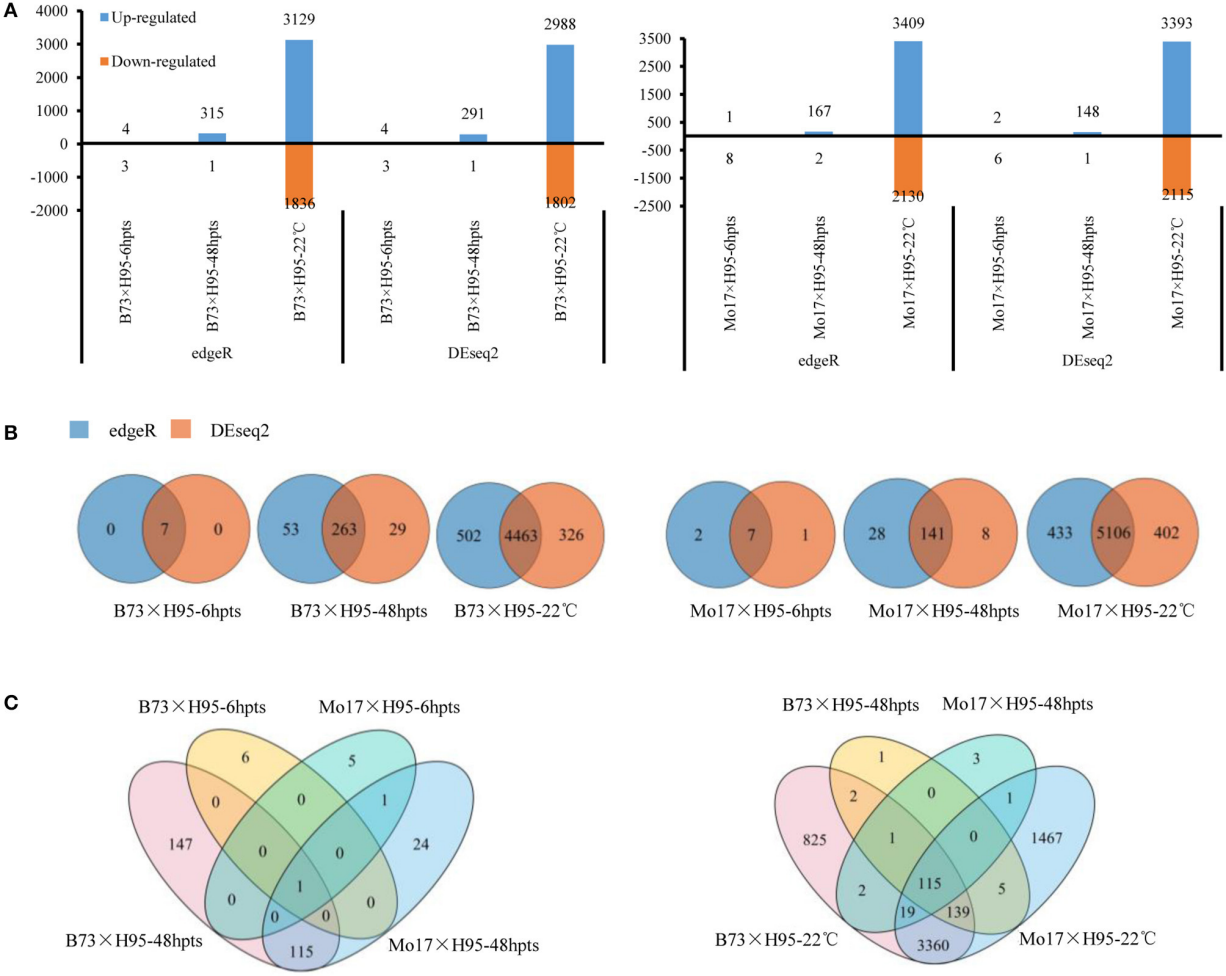
Differentially expressed genes (DEGs) in *Rp1-D21* mutant (mt) compared to the wild type (WT) in B73 × H95 and Mo17 × H95 backgrounds. **(A)** The up and downregulated DEGs in B73 × H95 and Mo17 × H95 backgrounds as calculated by edgeR and DEseq2. **(B)** Numbers of DEGs from edgeR and DEseq2 methods at different treatments. Venn diagrams of DEGs among different time points and temperature treatments from both edgeR and DEseq2. **(C)** The common DEGs between both genotypes at 6 and 48 h post temperature shift (hpts) (left), and at constant 22°C (right), respectively.

To facilitate the global identification of genes triggered during *Rp1-D21*-mediated hypersensitive response (HR), uniquely mapped DEGs were identified by comparing the gene expression levels and the abundance of each transcript in *Rp1-D21* mt relative to the WT for each near-isogenic pair at each timepoint. The DEGs were identified with a false discovery rate (FDR) or adjusted *p*-values (padj) < 0.05 and a |log2 (fold-change) |>1. Since we want to analyze the most robust DEGs, we detected DEGs using both edgeR and DEseq2 software, and only those DEGs identified by both the methods were used for further analysis. The number of DEGs detected by edgeR/DEseq2 methods at each timepoint/condition is shown in Figure 1A and Supplementary Table 2. In every case, the large majority of DEGs were identified by both the methods (Figure 1B). At 6 hpts, seven common DEGs were identified in B73 × H95 and Mo17 × H95 backgrounds using both the methods (Figure 1B). At 48 hpts, 263 and 141 common DEGs were identified in B73 × H95 and Mo17 × H95 backgrounds, respectively, and 116 genes were commonly identified in both the backgrounds (Figures 1B,C). In the constant temperature experiment, 4,463 (2,799 induced, 1,664 repressed) and 5,106 (3,156 induced, 1,950 repressed) DEGs were identified in B73 × H95 and Mo17 × H95 backgrounds, respectively (Figure 1B). Of these, 3,633 common DEGs were detected in both the backgrounds using two methods (Figure 1C).

Many more DEGs were identified in the constant temperature experiment (>5,000) than in the temperature shift experiment (149–316 at 48 hpts) in both the backgrounds. In every case, more DEGs were upregulated than downregulated by the presence or activation of Rp1-D21 (Figure 1A). There were some notable differences between the two genetic backgrounds. For example, at 48 hpts, the number of DEGs in the B73 × H95 background was almost twice that in the Mo17 × H95 background. On the other hand, substantially more DEGs, both up- and downregulated, were detected in the Mo17 × H95 background at constant 22°C.

### Functional Enrichment for *Rp1-D21*-Mediated DEGs

**Figure 2:**
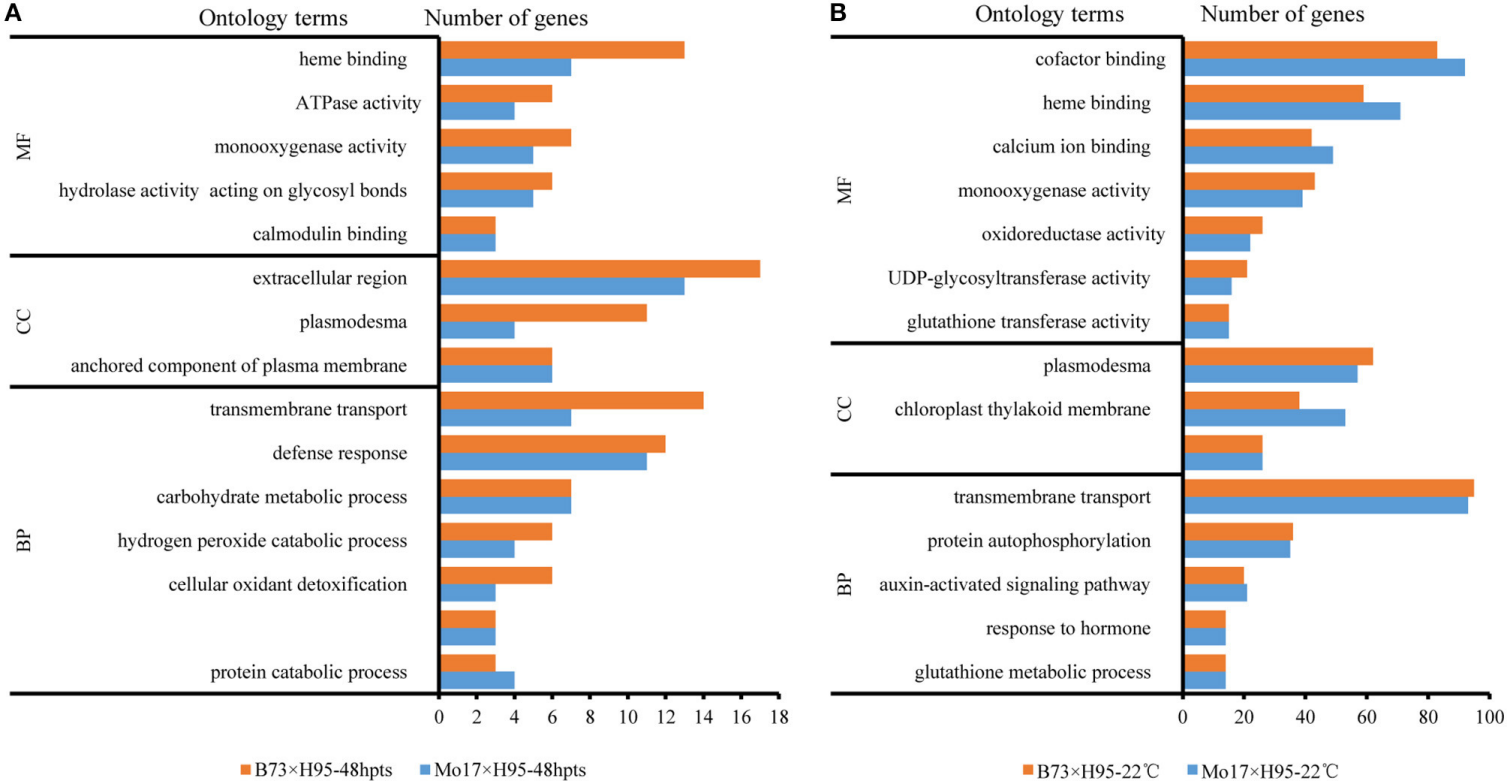
The Gene ontology (GO) enrichment of differentially expressed genes (DEGs) in maize *Rp1-D21* mutant (mt) compared to the wild type (WT) for the indicated times. **(A)** Top 15 GO subcategories of DEGs from *Rp1-D21* mt in B73 × H95 and Mo17 × H95 backgrounds at 48 hours post temperature shift (hpts). **(B)** Top 15 GO subcategories of DEGs from *Rp1-D21* mt in B73× H95 and Mo17 × H95 backgrounds at constant 22°C. MF, molecular function; CC, cellular component; BP, biological process.

The maize gene ontology (GO) database was used for the functional annotation of the DEGs identified by both edgeR and DEseq2 in B73 and Mo17 backgrounds. At 6 hpts, 6 out of 7 DEGs were predicted to be involved in adenosine diphosphate (ADP) binding (GO:0043531), and oxidation-reduction process (GO:0055114). At 48 hpts, the DEGs were mainly predicted to be involved in heme binding (GO:0020037), extracellular region (GO:0005576), transmembrane transport (GO:0055085), and defense response (GO:0006952), which included some genes in salicylic acid (SA) biosynthetic process (GO:0080142) (Figure 2A). At constant 22°C, the DEGs in the biological process category mainly included transmembrane transport, protein autophosphorylation, and hormone signaling pathway (Figure 2B). For molecular function category, the enriched GO terms mainly contained genes involved in binding (e.g., cofactor and heme binding) and catalytic activity [e.g., uridine diphosphate (UDP)-glycosyltransferase activity] (Figure 2B). For cellular component (CC) category, the enriched GO terms included plasmodesma, chloroplast thylakoid membrane, and intracellular component of the plasma membrane (Figure 2B).

Upon the initiation of HR, transcriptional activation genes were mainly involved in genes encoding receptor kinases, transcription factors, calcium regulation, and protein degradation (Supplementary Figure 4) as analyzed by MapMan software (Usadel et al., 2009). At constant 22°C conditions, a large number of transcription factors (TFs) were differentially expressed in *Rp1-D21* mts from both B73 × H95 and Mo17 × H95 backgrounds, indicating that massive transcription reprogramming occurred at this condition. Most of these genes were related to the establishment of transcriptional reprogramming and the enhancement of immune response for *Rp1-D21*-mediated HR. Gene regulatory networks (GRNs) represent the maps of potential transcriptional regulation between TFs and their target genes. To investigate the GRNs of Rp1-D21-mediated HR, the GRNs of the 3,633 DEGs detected in both B73 and Mo17 backgrounds at constant 22°C were predicted for the regulatory interactions with their upstream TFs based on the existing TF binding motifs and the conservation of TF binding sites (TFBSs) (Zhou et al., 2020). In total, 73 putative TFs were predicted to bind DEGs at constant 22°C condition under cutoff *p*-value ≤ 0.05 (Supplementary Table 3). Of those 73 TFs, the majority are WRKY (16, 21.92%), bZIP (14, 19.18%), MYB (9, 12.33%), and NAC (9, 12.33%). Some TFs were predicted to target themselves (Supplementary Table 3). Most of other DEGs which were related to biotic stresses, included genes involved in protein modification, protein degradation, calcium regulation, and hormone biosynthesis. Interestingly, many DEGs encoded receptor kinases, G-proteins, and mitogen activated protein (MAP) kinases, which directly or indirectly regulate the signal perception and activation of immunity, such as SA defense-related pathway.

### Many Genes Involved in PTI and SA Pathway Are Differentially Expressed in *Rp1-D21* Mutant

Pathogen-triggered immunity (PTI) and effector-triggered immunity (ETI) can function synergistically to protect plants against pathogens. Interestingly, we found that many genes predicted to be involved in maize PTI were differentially expressed in *Rp1-D21* mt, including the homologs of *BAK1, FLS2*, Ca^2+^-ATPase genes (*CA*s), and *RBOH*s (Supplementary Figure 5, Supplementary Table 4). Some key components predicted to act in the plant immunity signaling pathway were also differentially expressed, for instance, the mitogen-activated protein kinases (*MAPK*s) cascades and receptor-like cytoplasmic kinase VII (*RLCK VII*) subfamily genes, which act as central players in both PTI and ETI (Liang and Zhou, 2018; Zhou and Zhang, 2020). The SA pathway plays important roles in plant disease resistance and pathogen-induced HR (Zheng et al., 2015; Cui et al., 2017; Zhang et al., 2017). We found that the genes predicted to encode the SA receptors, *nonexpressor of pathogenesis-related genes 1* (*NPR1*) and *NPR4*, and the SA marker genes, *pathogenesis-related 1* (*PR1*) and *PR5* were significantly differentially expressed in *Rp1-D21* mt.

### The Differential Accumulated Metabolites and the Association With Transcriptomic Analysis in *Rp1-D21*

To investigate the metabolic changes induced by *Rp1-D21*, widely targeted metabolome analysis (Chen et al., 2013) was conducted by ultra-high-performance liquid chromatography-tandem mass spectroscopy (UPLC-MS). Due to the lack of seeds in B73 × H95 or Mo17 × H95 backgrounds, we used the isogenic hybrids A632 × H95-*Rp1-D21* and A632 × H95 for this experiment. A total of 423 metabolites were detected, including lipid, phenolic acids, flavonoids, and alkaloids. The orthogonal partial least squares discriminant analysis (OPLS-DA) model was performed on the metabolic analysis, and significantly differential accumulated metabolites (DAMs) were selected with |log2 (fold-change)| > 0.6 and variable importance for projection (VIP) > 1. Finally, a total of 103 DAMs (76 upregulated and 27 downregulated in the *Rp1-D21* background) were identified (Supplementary Figure 6A, Supplementary Table 5). Pathway enrichment analysis was conducted using Kyoto encyclopedia of genes and genomes (KEGG). These DAMs were mainly divided into “biosynthesis of secondary metabolites,” “biosynthesis of amino acids,” and “phenylpropanoid biosynthesis” (Supplementary Figure 6B).

**Figure 3:**
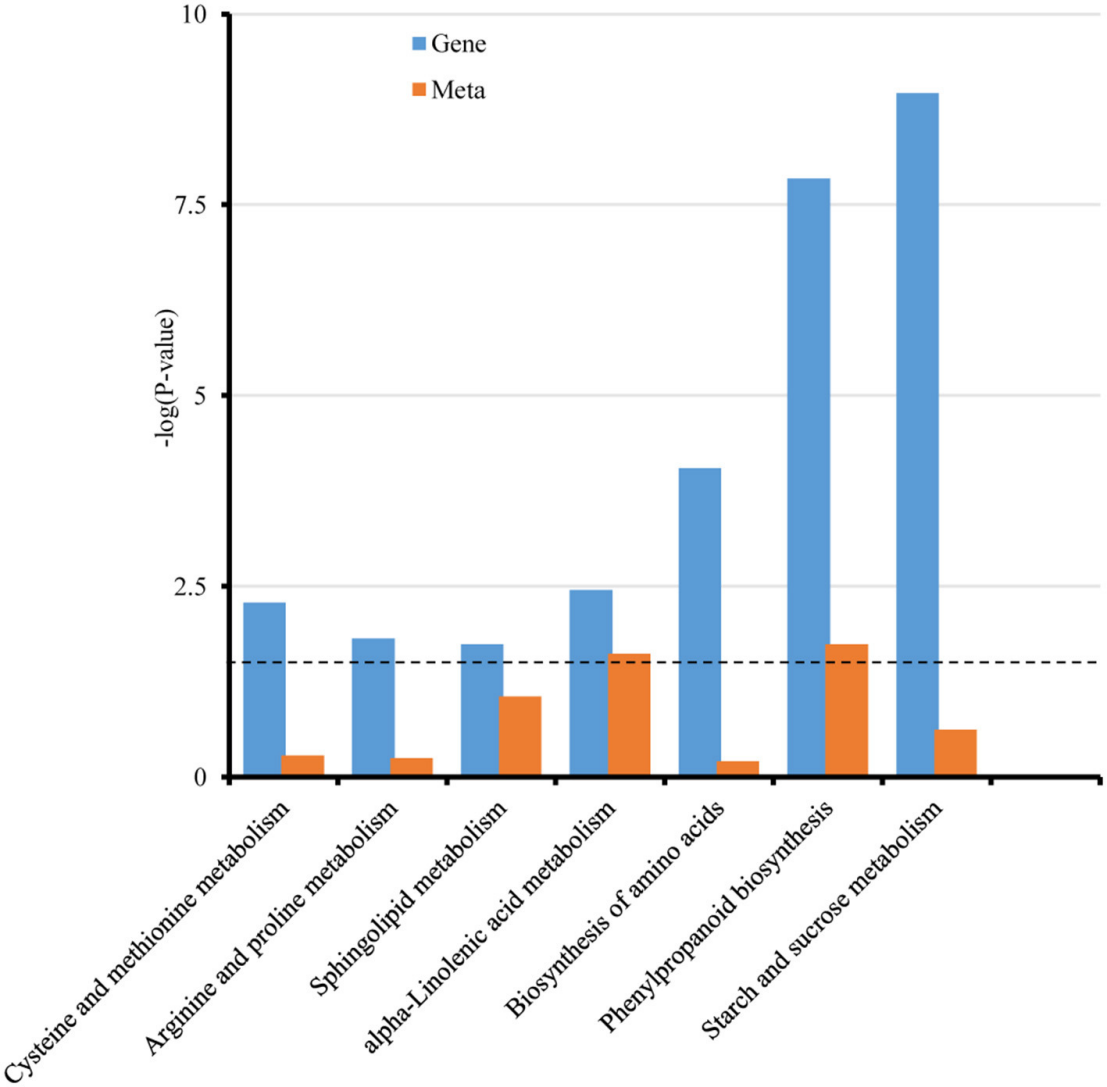
Integrated analyses of transcriptomic and metabonomic results of *Rp1-D21* mutant (mt) compared to the wild type (WT). The Kyoto Encyclopedia of Genes and Genomes (KEGG) enrichment histogram of conjoint analysis of DEGs and differential accumulated metabolites (DAMs). The x-axis represents the metabolic pathways and the y-axis represents the expression as -log (*p*-value). The blue columns represent the enrichment *p*-values of DEGs, and the orange columns represent the enrichment *p*-values of DAMs. The black dotted line represents the threshold for significant enrichment at *p* < 0.05.

To study the association between transcriptomic and metabolic analyses in *Rp1-D21* mt, the conjoint analysis of DAMs and DEGs at constant temperature were conducted by the KEGG pathway. Seven common pathways were enriched in *Rp1-D21* mt (Figure 3, Supplementary Table 5), with phenylpropanoid biosynthesis (Supplementary Figure 7) and α-linolenic acid metabolism as the top two significantly enriched pathways.

### SA Pathway Genes and Metabolites Are Differentially Accumulated in *Rp1-D21*

**Figure 4:**
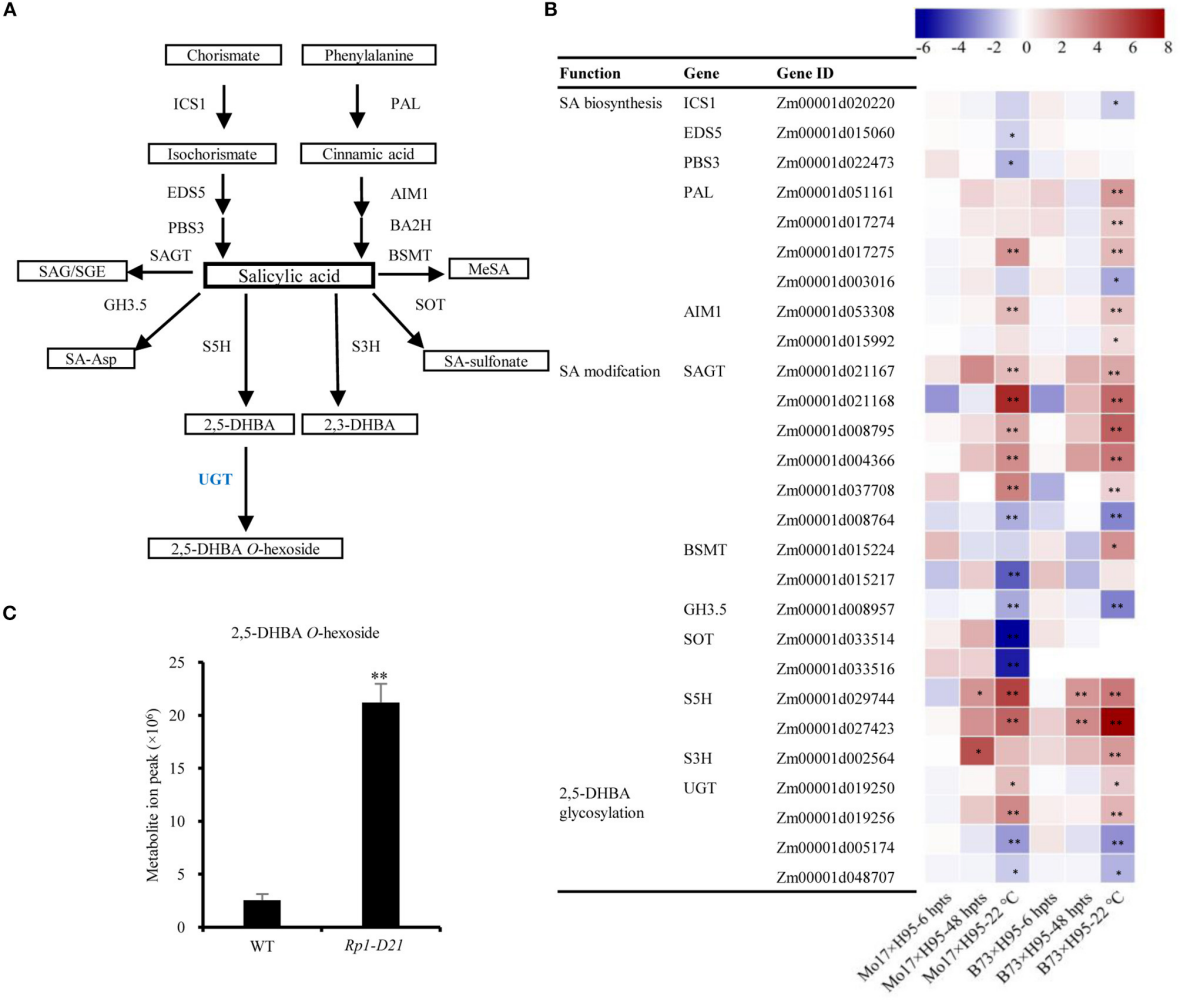
The key enzymes involved in salicylic acid (SA) biosynthesis and modification pathways. **(A)** The SA biosynthesis and modification pathways. ICS1, isochorismate synthase 1; PAL, phenylalanine ammonia-lyase; EDS5, enhanced disease susceptibility 5; PBS3, AvrPphB susceptible3; AIM1, abnormal inflorescence meristem1; BA2H, benzoic acid 2-hydroxylase; UGT, UDP-glucosyltransferases; SAGT, salicylic acid glucosyltransferase; SAG, SA 2-O-β-Dglucoside; SGE, salicylate glucose ester; BSMT, benzoic acid/salicylic acid carboxyl methyltransferase; MeSA, methyl salicyliate; GH3.5, GH3 acyl adenylase family member 3.5; SA-Asp, salicyloyl-L-aspartate; SOT, sulfotransferase; S5H, SA 5-hydroxylase; S3H, SA 3-hydroxylase; 2,5-DHBA, 2,5-dihydroxybenzoic acid; 2,3-DHBA, 2,3-dihydroxybenzoic acid. **(B)** The log2 fold-change of the genes encoding key enzymes in SA biosynthesis and modification pathways. The fold change was normalized with the FPKM value. **(C)** The levels of 2,5-DHBA *O*-hexoside were significantly accumulated in *Rp1-D21* mutant (mt) compared to the wild type (WT). Asterisks indicate significant difference between samples, each sample with three biological replicates.

Salicylic acid and its derivatives play important roles in plant disease resistance (Vlot et al., 2009; Ding et al., 2018); therefore, we investigated the DEGs and DAMs in the SA pathway. The SA biosynthesis occurs *via* two pathways, the isochorismate synthase (ICS) pathway and the phenylalanine ammonia-lyase (PAL) pathway, which play the major and minor roles in SA biosynthesis, respectively (Figure 4A, Dempsey et al., 2011). Interestingly, most genes encoding enzymes in ICS biosynthesis, including ICS1, EDS5, and PBS3, were downregulated in the late stage of HR induction (22°C), while most genes in the PAL pathway had an opposite expression pattern, including PAL and AIM1 homologs (Figure 4B, Supplementary Table 6). The level of phenylalanine, the precursor of the PAL pathway was increased to 1.53 fold in *Rp1-D21* mt compared to WT (Supplementary Table 5). In addition to the SA biosynthesis, the regulation of genes predicted to be involved in SA modifications were also investigated (Figure 4B). The genes associated with SA hydroxylation (S5H and S3H) were highly induced, while the genes related to SA methylation (BSMT), amino acid conjugation (GH3.5), and SA sulfonation (SOT) were downregulated in the late stage of HR (Figures 4A,B). In Arabidopsis, AtUGT74F1/2 and AtUGT75B1 modify SA to generate glycosylated SA (Noutoshi et al., 2012; George Thompson et al., 2017). We identified 9 and 4 SA glucosyltransferase (SAGT) homologs when searched the maize genome for sequences homologous to AtUGT74F1/2 and AtUGT75B1, respectively (Supplementary Figure 8). Among these 13 genes encoding SAGTs, 5 and 1 were up- and downregulated in *Rp1-D21*, respectively (Figure 4B, Supplementary Figure 8).

**Figure 5:**
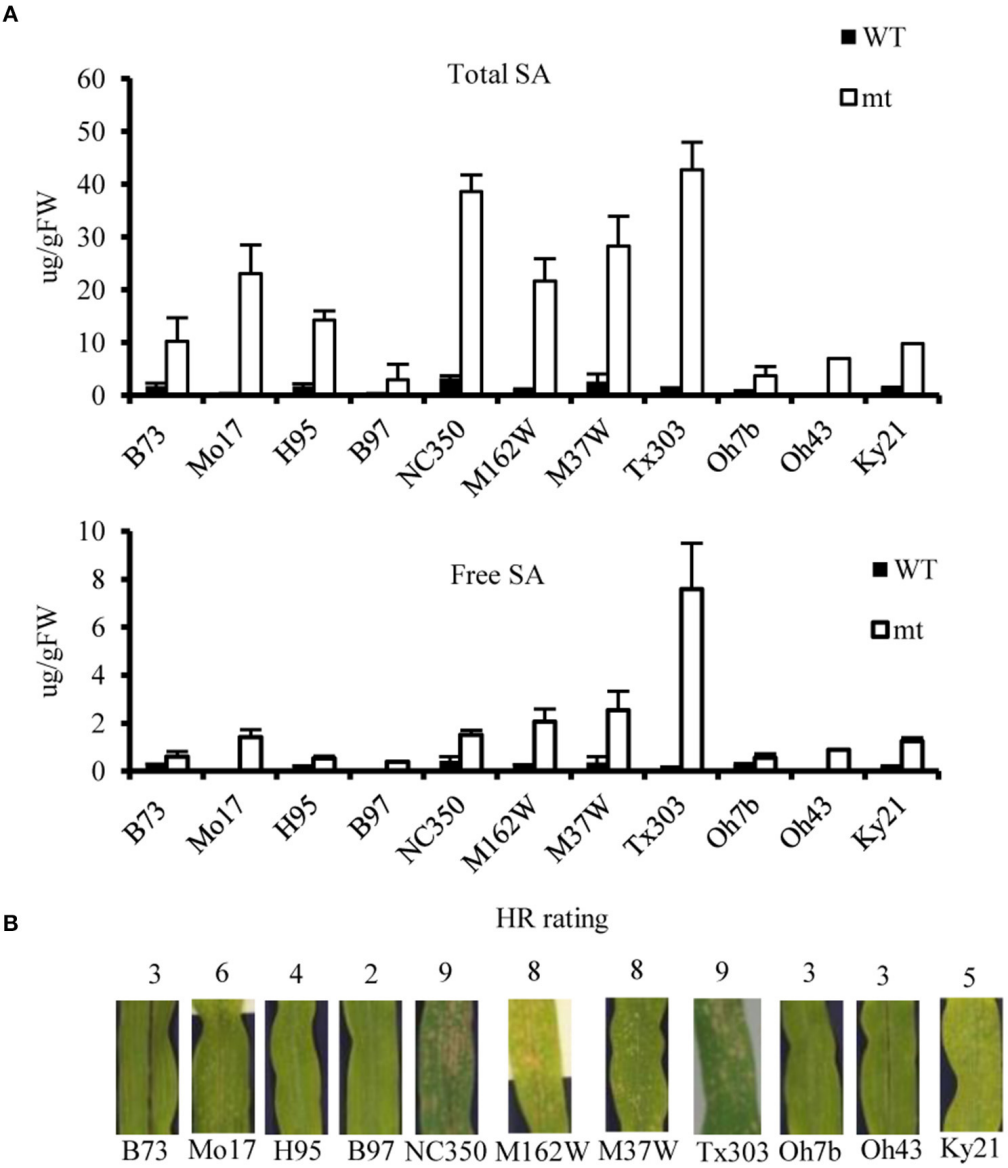
The salicylic acid (SA) levels and hypersensitive response (HR) severity of *Rp1-D21* in different genetic backgrounds. **(A)** The levels of total SA and free SA were significantly accumulated in *Rp1-D21* mutant (mt) compared to the wild type (WT) in different backgrounds crossed with H95-*Rp1-D21* when they were grown at 22°C. **(B)** The HR rating of *Rp1-D21* mt in different backgrounds crossed with H95-*Rp1-D21*grown at 22°C condition.

**Figure 6:**
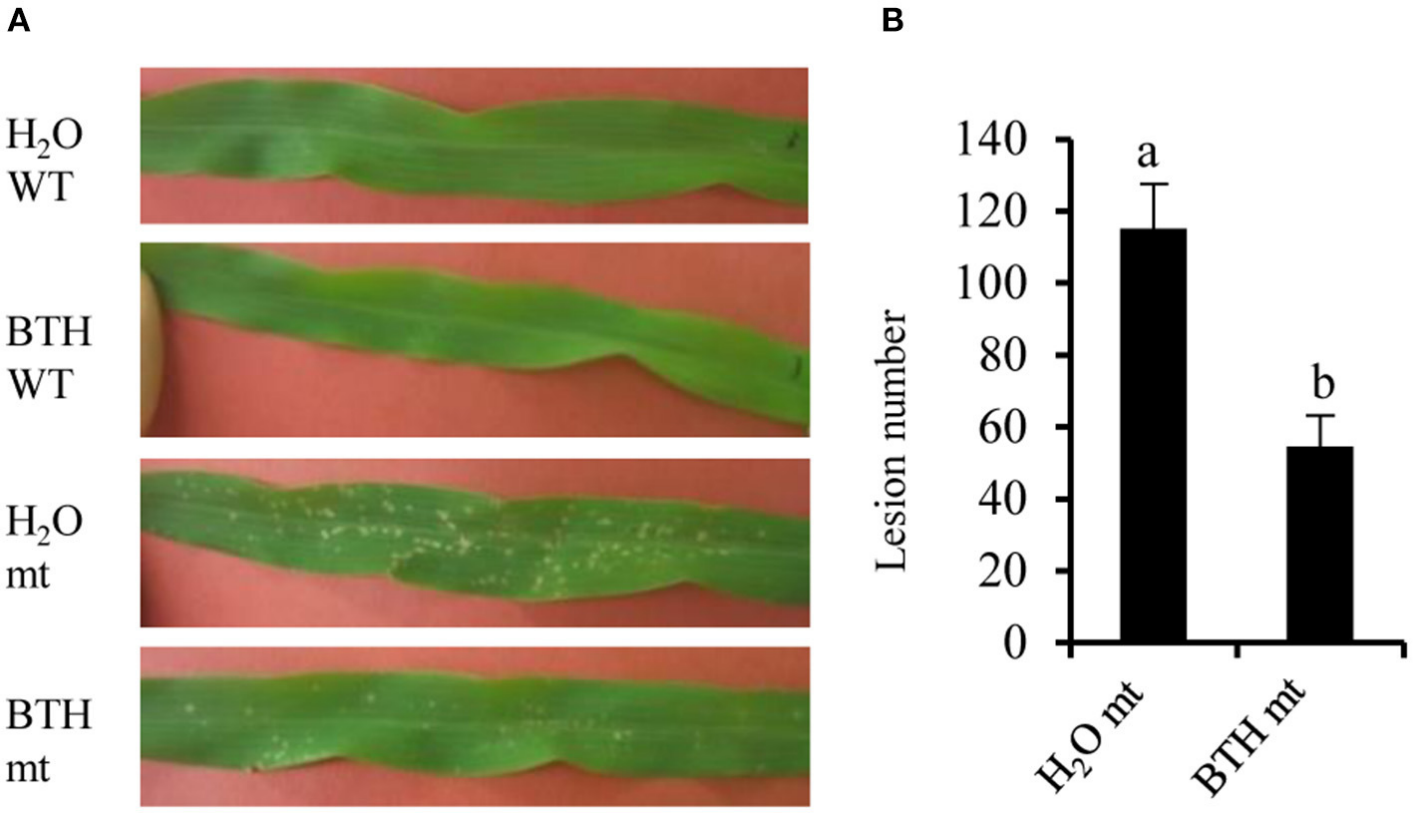
Benzothiadiazole (BTH) treatment reduced Rp1-D21-mediated HR. **(A)** Plants in Mo17 × H95 background were grown at 30°C for 8 days, then the plants were treated by H_2_O or 1.2 mM BTH. After treatment, the temperature was dropped to 22°C to induce hypersensitive response (HR), and the pictures of the third leaves were taken at 4 days after temperature shift. **(B)** The lesion number in mutant (mt was calculated after H_2_O and BTH treatment. Significant differences (*p* < 0.05) between samples are indicated by different letters (a,b). Wild type (WT) and *Rp1-D21* mutant (mt).

Salicylic acid can be hydroxylated by S5H to form 2,5-dihydroxy benzoic acid (2,5-DHBA), which can be further glycosylated by UGT76D1 to generate 2,5-DHBA glucosides in Arabidopsis (Zhang et al., 2017; Huang et al., 2018). We further measured the total and free SA levels of H95-*Rp1-D21* crossed into different backgrounds when they were grown at 22°C, and the result showed that *Rp1-D21* mt in different backgrounds had more SA accumulation than their corresponding WT (Figure 5A). Interestingly, the SA levels were largely positively related with the HR strength in different backgrounds (Figure 5B). To investigate whether SA affect Rp1-D21-mediated HR, we grew Mo17x H95-*Rp1-D21* at 30°C for 8 days, and then treated the seedlings with H_2_O and 1.2 mM benzothiadiazole S-methyl ester (BTH), an analog of SA, and put the plant at 22°C to induce HR. We found that BTH significantly reduced the severity of *Rp1-D21*-mediated HR compared to H_2_O (Figure 6). Among the DAMs, 2,5-DHBA *O*-hexoside was identified as one of the top DAMs, which accumulated at 8.34-fold in *Rp1-D21* mt than the WT (Figure 4C, Supplementary Table 5).

### DHBA Glucosyltransferases (*ZmUGTs*) Are Induced in *Rp1-D21*

**Figure 7:**
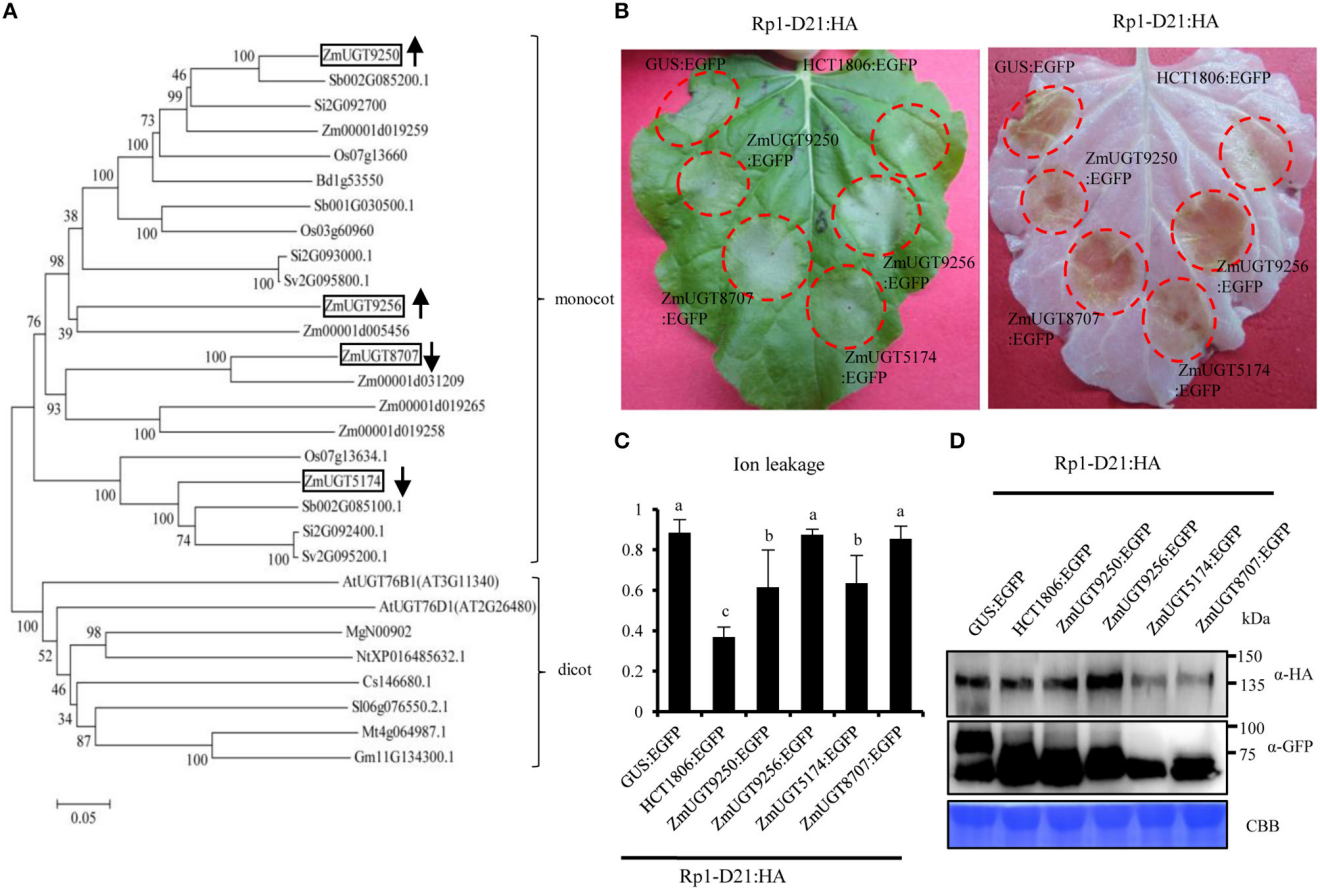
Phylogenetic analysis of UDP-glucuronosyltransferase (UGT) proteins and the function of ZmUDP-glucuronosyltransferases (UGTs) in Rp1-D21-mediated hypersensitive response (HR). **(A)** The phylogenetic tree was constructed using MEGA 6.0 software. At, *Arabidopsis thaliana*; Bd, *Brachypodiumdistachyon*; Cs, *Cucumissativus*; Gm, *Glycine max*; Mg, *Mimulusguttatus*; Mt, *Medicagotruncatula*; Nt, *Nicotianatabacum*; Os, *Oryza sativa*; Sb, *Sorghum bicolor*; Si, *Setaria italica*; Sl, *Solanum lycopersicum*; Sv, *Setaria viridis*; Zm, *Zea mays*. The boxes indicate the UGT homologs of maize which were differentially expressed in *Rp1-D21*. **(B)** ZmUGTs were transiently co-expressed with Rp1-D21 into *Nicotiana benthamiana*. The representative leaf was photographed at 3 days after inoculation (left), and the same leaf was cleared by ethanol (right). **(C)** Ion leakage conductivity [average ± standard error (SE), *n* > 5] was measured at 60 h after co-expression of GUS, HCT1806, or ZmUGTs with Rp1-D21. Significant differences (*p* < 0.05) between samples are indicated by different letters (a–c). **(D)** Total protein was extracted from agro-infiltrated leaves at 40 hour post inoculation (hpi). Anti-HA was used to detect the expression of Rp1-D21, and anti-GFP was used to detect the expression of GUS, HCT1806, and ZmUGTs. Equal loading of protein samples was shown by Coomassie brilliant blue (CBB) staining. The experiments were performed three times with similar results.

In Arabidopsis, AtUGT76D1 (AT2G26480) is responsible for the conversion from 2,5-DHBA to 2,5-DHBA glucoside (Huang et al., 2018). We identified UDP-glucuronosyltransferase (UGT) homologs from maize (ZmUGTs) according to AtUGT76D1 and performed a phylogenetic analysis (Figure 7A). Of the nine ZmUGT homologs identified, Zm00001d019250 (ZmUGT9250) and Zm00001d019256 (ZmUGT9256) were upregulated at the late stage of HR induction in the *Rp1-D21* mt compared to the corresponding WT in both B73 and Mo17 backgrounds (Figure 7A), while Zm00001d005174 (ZmUGT5174) and Zm00001d048707 (ZmUGT8707) had the opposite expression pattern (Supplementary Table 7). Other ZmUGT homologs were not differentially expressed under these conditions (Supplementary Table 7).

### ZmUGTs Partially Suppress Rp1-D21-Mediated HR in *Nicotiana benthamiana*

To investigate the possible roles of the four differentially regulated ZmUGTs in Rp1-D21-mediated HR, we used the agrobacteria-mediated transient expression in *Nicotiana benthamiana*. The four ZmUGTs were fused with a C-terminal enhanced green fluorescent protein (EGFP) tag. GUS:EGFP and HCT1806:EGFP were used as negative and positive controls, respectively. The HCT1806 had previously been shown to be a strong suppressor of Rp1-D21-mediated HR (Wang et al., 2015b; Wang and Balint-Kurti, 2016; Murphree et al., 2020). When transiently co-expressed with Rp1-D21 fused with C-terminal 3 × hemagglutinin (HA) tag in *N. benthamiana*, ZmUGT9250 and ZmUGT5174 partially suppressed Rp1-D21-induced HR compared to HCT1806, while ZmUGT9256 and ZmUGT8707 did not suppress Rp1-D21-induced HR (Figure 7B). Ion leakage conductivity data further verified our visual observations (Figure 7C). Co-expression of ZmUGTs did not significantly change the protein accumulation of Rp1-D21 which was expressed at substantial and broadly comparable levels (Figure 7D).

The N-terminal coiled-coil (CC) domain of Rp1-D21 protein (CC_D21_) conferred an obvious autoactive HR when it was fused with EGFP and transiently expressed in *N. benthamiana* (Wang et al., 2015b). To determine whether ZmUGTs can suppress CC_D21_-mediated HR, we co-expressed them and CC_D21_:EGFP in *N. benthamiana*. The results showed that ZmUGT9250 suppressed CC_D21_-mediated HR which was similar to HCT1806, ZmUGT5174 partially suppressed CC_D21_-mediated HR, and ZmUGT9256 and ZmUGT8707 had no obvious effect (Supplementary Figure 9).

### ZmUGTs Have No Interaction With CC_D21_

Several Rp1-D21 regulators we identified previously interact with CC_D21_ and suppress CC_D21_-mediated HR (Wang et al., 2015b; Wang and Balint-Kurti, 2016; Zhu et al., 2020; Liu et al., 2021; Luan et al., 2021). To investigate whether ZmUGTs can interact with CC_D21_, we performed yeast two-hybrid assays. We did not detect interaction between any of the four ZmUGTs investigated and CC_D21_ (Supplementary Figures 10A,B). A co-immunoprecipitation assay between CC_D21_ and ZmUGT9250, which had the strongest suppression effect on CC_D21_-mediated HR, also did not detect any interaction (Supplementary Figure 10C).

### ZmUGTs Have No Obvious Suppression Effect on Other Elicitor-Induced HR

To investigate whether ZmUGTs could suppress HR induced by other nucleotide-binding leucine-rich-repeats (NLRs), we co-expressed them with *Arabidopsis* RPM1(D505V) and barley MLA10(D502V), which confer an autoactive HR when transiently expressed in *N. benthamiana* (Gao et al., 2011; Bai et al., 2012). The results showed that all the four ZmUGTs had no obvious suppression effect on either MLA10(D502V)- or RPM1(D505V)-induced HR (Supplementary Figure 11). We further co-expressed ZmUGTs with BAX or INF, the death-promoting protein or cell death elicitor (Kamoun et al., 1998; Lacomme and Santa Cruz, 1999), and found that neither of them substantially suppressed either BAX- or INF-induced HR (Supplementary Figure 11).

## Discussion

In this study, we used transcriptome and metabolome analyses to investigate the molecular responses of Rp1-D21-mediated hypersensitive response (HR). The conditions used for the transcriptome analysis and metabolome analysis differed substantially in terms of the growth conditions and the genotypes used. Despite this, the salicylic acid (SA) pathway and the phenylpropanoid biosynthesis pathway were induced at both the transcriptional and metabolic levels. Especially, the genes predicted to encode 2,5-DHBA UGTs were differentially expressed in *Rp1-D21* mutant (mt) and the 2,5-DHBA *O*-hexoside catalyzed by UGTs were high accumulated in *Rp1-D21* mt. We further examined that two Zm UDP-glucuronosyltransferases (UGTs) partially suppress Rp1-D21-mediated HR.

### The Autoactive *Rp1-D21* mt Is an Excellent Tool for the Identification of Disease Resistance Genes in Maize

Autoimmune mutants caused by inappropriate activation of nucleotide-binding leucine-rich-repeats (NLRs) are widely used for mechanistic investigation of immune signaling components in different plants, e.g., Arabidopsis *snc1* and *mkk1mkk2* (Kong et al., 2012; Dong et al., 2016; Chakraborty et al., 2018). Rp1-D21 is an NLR protein which confers autoimmunity in maize (Sun et al., 2001; Smith et al., 2010). The transcript levels of *Rp1-D21* were greatly increased over time after temperature dropping from 30 to 22°C, especially at 48 h post the temperature shift (hpts) (Supplementary Figure 2). At constant 22°C, the transcript levels of *Rp1-D21* were even much higher (Supplementary Figure 2). These data suggested that the onset of HR might be related with the increased expression of *Rp1-D21*. Consistent with the trends of the transcript levels of *Rp1-D21*, more differentially expressed genes (DEGs) were identified at constant 22°C than at 48 and 6 hpts, indicating that these DEGs were induced by the increased levels of *Rp1-D21*. Several transcription factors (TF)s, including WRKY and bZIP which are known to act in plant immunity (Jakoby et al., 2002; Kaminaka et al., 2006; Pandey and Somssich, 2009), were predicted as the major TFs to bind the promoters of DEGs (Supplementary Table 3), indicating that Rp1-D21-mediated HR is under tight transcriptional regulation. We showed that many genes predicted to act in pathogen-triggered immunity (PTI) were differentially expressed in the defense response triggered by the NLR Rp1-D21, including gene homologs encoding FLS2, BAK1, RBOHs, RLCKs, and MAPK cascades (Supplementary Figure 5). Consistent with our results, it was recently reported that effector-triggered immunity (ETI) can increase the defense strength through transcriptional induction of PTI signaling components and PTI is required for disease resistance mediated by several NLRs (Ngou et al., 2021; Yuan et al., 2021).

Several studies have used the *Rp1-D21* mt to identify genes or loci associated with modulation of the severity of HR (Chintamanani et al., 2010; Chaikam et al., 2011; Olukolu et al., 2014). We have verified that two homolog genes encoding enzymes of lignin biosynthesis pathway ZmHCT1806 and ZmCCoAOMT2 (Wang et al., 2015b; Wang and Balint-Kurti, 2016), two flavone synthase homologs of ZmFNSIs (Zhu et al., 2020), the nicotinate *N*-methyl transferase ZmNA*N*MT(Liu et al., 2021), and the metacaspase homologs, ZmMC1 and ZmMC2 (Luan et al., 2021), can suppress Rp1-D21-mediated HR likely through physical interaction with the cellular component (CC) signaling domain of Rp1-D21 when transiently expressed in *Nicotiana benthamiana*. Interestingly, ZmCCoAOMT2 was also proved to confer quantitative resistance to both southern leaf blight and gray leaf spot in maize (Yang et al., 2017). The homologs of these genes in different plant species also act in plant immunity (Coll et al., 2010; Senthil-Kumar et al., 2010; Gallego-Giraldo et al., 2011; Zeilmaker et al., 2015), suggesting the functional association between disease resistance and HR regulation. Recently, it was reported that the transcriptional responses triggered by Rp1-D21 are broadly similar to those triggered by its wild type (WT) counterpart Rp1-D, which is activated by *Puccinia sorghi* infection (Kim et al., 2021). The DEGs identified in *Rp1-D21* are highly correlated with disease resistance; therefore, *Rp1-D21* can be used as a quick and effective tool for exploring new HR regulation and disease resistance genes.

### Rp1-D21-Mediated HR Related Signal Transduction and Metabolic Pathways in Maize

Except for the SA pathway, we found that the α-linolenic acid pathways and the phenylpropanoid biosynthesis pathway were induced in *Rp1-D21* at both the transcriptional and metabolic levels according to the integrated transcriptome and metabolome analyses (Figure 3). The α-linolenic acid is produced by two metabolic pathways, 9-lipoxygenase (9-LOX) pathway, and 13-lipoxygenase (13-LOX) pathway (Wasternack, 2007). LOX-derived oxylipins have been implicated in plant growth and development, senescence, and resistance to pathogens (Blée, 2002; Mosblech et al., 2009). When infected by different pathogens, the transcript levels and the related metabolites in 9-LOX pathway were increased in tobacco, potato, and maize with the occurrence of HR (Gobel et al., 2001, 2003; Davoine et al., 2006; Gao et al., 2009). The 9-hydroxyoctadecatrienoic acid (9-HOT) and 9,10-epoxyoctadecenoic acid (9,10-EOT) in the 9-LOX pathways were among the most active oxylipins (Prost et al., 2005; Vicente et al., 2012). The mutant insensitive to 9-HOT displays enhanced susceptibility to *Pseudomonas* infection (Vellosillo et al., 2007). In our metabolites analysis, we found that the transcript levels of genes and metabolite products in the α-linolenic acid metabolism pathway were increased in the *Rp1-D21* mutant (Figure 3, Supplementary Table 5). In particular, we observed a massive accumulation of 9-HOT and 9,10-EOToxylipins derived from the 9-LOX pathway suggested that they play pivotal roles in Rp1-D21-mediated HR and plant disease resistance.

The phenylpropaniod biosynthesis pathway, mainly including lignin intermediates, anthocyanins, isoflavonoidphytoalexins, and phenolic compounds, has been implicated as one of the major pathways for defense against pathogens (Ranjan et al., 2019). As displayed in Figure 3, transcriptome and metabolome conjoint analysis indicated that differentially expressed genes (DEGs) and differential accumulated metabolites (DAMs) associated with the phenylpropaniod pathway were significantly enriched (Supplementary Table 5). Many DEGs in the lignin biosynthesis pathway, including *PAL*, cinnamoyl-CoA reductase (*CCR*), *CCoAOMT*, and *HCT* play important roles in plant defense response (Kawasaki et al., 2006; Gallego-Giraldo et al., 2011; Wang and Balint-Kurti, 2016; He et al., 2020). Many genes involved in phenylpropanoid pathway are differentially expressed after inoculation with the pathogen *Fusarium graminearum* (Liu et al., 2016). These results suggest that the phenylpropaniod biosynthesis pathway constituted a key class of enzymes and metabolites associated with modulating the HR induced by Rp1-D21 and disease resistance in maize defense response.

### SA and Glycosylation of SA Derivatives May Play a Role in Defense Response

We have previously shown that several regulators interact with CC_D21_ and modulate Rp1-D21-mediated HR (Wang et al., 2015b; Wang and Balint-Kurti, 2016; Zhu et al., 2020; Liu et al., 2021; Luan et al., 2021). Here we found that ZmUGTs suppressed Rp1-D21- and CC_D21_-mediated HR, but they did not interact with CC_D21_ (Supplementary Figure 10). These data suggested that the regulation of Rp1-D21-mediated HR by ZmUGTs might not be through the formation of a protein complex with Rp1-D21.

The plant hormone, SA has profound importance in both local resistance against biotrophic and hemi-biotrophic pathogens and systemic acquired resistance (Vlot et al., 2009). In local pathogen infection, high levels of SA induce defense gene expression by activating the transcriptional activator NPR1 and lead to cell death (Fu and Dong, 2013). In the maize lesion mimic mutant, *Les4*, the free and total SA levels were significantly increased at the lesion-developed stage compared to the prelesion stage (Morris et al., 1998). In this study, many gene homologs in SA biosynthesis and metabolism pathways were differentially expressed in *Rp1-D21* (Figure 4B). Interestingly, the genes in the isochorismate synthase 1 (ICS1) and phenylalanine ammonia-lyase (PAL) pathways were mostly down- and upregulated in *Rp1-D21* mt compared to WT at the late stage, respectively (Figure 4B). The level of phenylalanine was also increased in *Rp1-D21* mt compared to WT (Supplementary Table 5). However, cinnamic acid, chorismate, and isochorismate were not identified under our conditions. The levels of total SA and free SA were significantly elevated in *Rp1-D21* mts compared to the corresponding WT and there was a positive correlation between SA levels and the HR strength in different backgrounds (Figure 5). These data suggested that the activation of the SA pathway might be associated with the formation of HR lesions in plants carrying *Rp1-D21*. The upregulation of genes in the PAL pathway might act to keep the SA level at an appropriate level to trigger HR while the downregulation of genes in the ICS1 pathway might act to prevent the over-accumulation of SA at the late stage. On the other hand, the exogenous application of SA or SA analogs suppresses ETI-mediated HR in Arabidopsis (Zavaliev et al., 2020). Similarly, we found that exogenous application of benzothiadiazole (BTH), an analog of SA, also suppressed Rp1-D21-mediated HR in maize (Figure 6). These studies suggest that SA plays important roles in suppressing NLR-mediated HR. Therefore, SA plays dual roles in HR regulation, likely through the induction of positive defense regulators to activate HR or induction of negative regulators to suppress HR (Radojicic et al., 2018; Peng et al., 2021).

Excessive levels of SA can be toxic to plants; therefore SA can be modified by different conjugations to less toxic derivatives (Dempsey et al., 2011). In Arabidopsis, two SAGTs, UGT74F1, and UGT76B1 can glycosylate SA to produce SAG (Noutoshi et al., 2012). Loss of UGT74F1 and UGT76B1 confers resistance to both *Pst* DC3000 and a strain which can be recognized by the NLR protein RPM1 (Noutoshi et al., 2012). Here we found that several maize SAGT homologs were upregulated in *Rp1-D21* and the total SA levels were increased in *Rp1-D21* (Figures 4, 5), suggesting that these SAGT homologs might play a role in Rp1-D21-mediated HR.

2,5-dihydroxybenzoic acid (2,5-DHBA) and 2,3-DHBA are two major catabolic products of SA and they are catalyzed by hydroxylating SA *via* S5H and S3H, respectively (Figure 4B, Zhang et al., 2017). 2,5-DHBA is widely distributed in planta and is induced by the pathogen, *Pst*DC3000 in *Arabidopsis* (Zhang et al., 2017). In a previous study (Zhu et al., 2020), we found that the transcript levels of *ZmFNSI*s (also known as *ZmS5H*s) and *ZmS3H* were upregulated in *Rp1-D21* mt (Figure 4B), and ZmS5Hs but ZmS3H does not suppress Rp1-D21- and CC_D21_-mediated HR (Zhu et al., 2020), indicating that ZmS5Hs play important roles in maize defense response. The 2,5-DHBA and 2,3-DHBA can be further glycosylated by a unique UDP-glycosyltransferase UGT76D1 in Arabidopsis (Huang et al., 2018). Expression of the *AtUGT76D1* is induced by *Pst* DC3000. Overexpression of *AtUGT76D1* leads to high SA accumulation, upregulation of defense genes and the autoactive HR phenotype, while the immune responses were compromised when *AtUGT76D1* is knocked out implying that AtUGT76D1 is a positive regulator in plant innate immunity (Huang et al., 2018). Interestingly, AtUGT76B1, a close homolog which had 38.8% similarity of amino acid with AtUGT76D1, was recently found to use *N*-hydroxy-pipecolic acid (NHP) as substrate and act in systemic-acquired resistance (Bauer et al., 2021; Holmes et al., 2021; Mohnike et al., 2021). Loss-of-function mts of *AtUGT76B1* confer a dwarf phenotype and constitutive defense response, with high NHP and SA accumulation and enhanced disease resistance to *Pst*DC3000 (Bauer et al., 2021; Mohnike et al., 2021). The 2,5-DHBA glucosides were also accumulated in tomato and cucumber after infection with the pathogen, citrus exocortis viroid (CEVd) and prunus necrotic ringspot virus (PNRSV) (isolate NCM1), respectively (Fayos et al., 2006). In maize, 147 UGTs belonging to 17 groups were identified (Li et al., 2014), and none of them have been functionally investigated in disease resistance. Here we also found that the transcript levels of four DHBA-glycosyltransferase *ZmUGT* homologs of AtUGT76D1 were significantly differentially expressed and higher levels of SA and 2,5-DHBA *O*-hexoside were accumulated in *Rp1-D21* mt compared to WT (Figures 4, 5, 7). We further showed that the two ZmUGTs, ZmUGT9250 and ZmUGT5174, suppressed Rp1-D21-mediated HR. These results indicated that ZmUGTs play negative roles in NLR protein Rp1-D21-mediated defense response, which is different from the positive role of AtUGT76D1 in plant immunity. Interestingly, ZmUGT9250 and ZmUGT5174 have opposite expression patterns in the lines harboring Rp1-D21 (Figures 4, 7). The transcript level of ZmUGT9250 was increased in Rp1-D21, suggesting that it might mainly act to inhibit the further spreading of HR when Rp1-D21 is activated, while the transcript level of ZmUGT5174 was decreased in Rp1-D21, suggesting that it might mainly act to keep NLR protein in the inhibited state in WT. The two ZmUGTs had no obvious effects on RPM1 (D505V), MLA (D502V), INF1, or Bax-mediated cell death (Supplementary Figure 11), indicating that they are not general cell death suppressors and they might have different mechanisms with Rp1-D21 for triggering cell death. It was reported that DHBA glycosyltransferases play important roles in the innate immune response through regulating the SA homeostasis (Huang et al., 2018). In this way, we hypothesized that the accumulation of DHBA glycosides which might act as an endogenous modulator of SA levels to switch the activity of enzymes in SA-mediated signaling pathways, thus regulating the HR phenotype in *Rp1-D21*. Since the enzyme activity of the four ZmUGThomologs has not been investigated yet, it is not clear which member has the DHBA glycosyltransferase activity. Since these ZmUGTs were homologous to both AtUGT76D1 and AtUGT76B1 (Figure 7A), it will be interesting to further investigate whether these ZmUGTs are also functionally similar to AtUGT76B1.

In summary, we used transcriptomic and metabolomic analyses to identify DEGs and DAMs involved in Rp1-D21-meidiated HR, which provides a useful resource for exploring maize disease resistance genes. The SA metabolic pathway and the phenylpropanoid biosynthesis pathway were induced at both the transcriptional and metabolic levels. We further demonstrated that two of four *ZmUGTs* partially suppressed the HR triggered by Rp1-D21 or its N-terminal CC_D21_ domain, which forms the basis for further investigating their roles in plant immunity.

## Experimental Procedures

### Plant Materials and Growth Conditions

Maize (*Zea mays*) line B73 was used for isolating *ZmUGTs*. The *Rp1-D21*-H95 line was generated by repeatedly backcrossing a line carrying Rp1-D21 as a female to the H95 inbred. As previously reported (Chaikam et al., 2011), *Rp1-D21* mutant is maintained in the heterozygous state in H95 background (H95-*Rp1-D21*) due to its sterile nature in the homozygous state. The H95-*Rp1-D21* line was then crossed as female to B73 and Mo17 to produce the isogenic hybrid pairs, B73 × H95 and B73 × H95*-Rp1-D21* and the isogenic pair, Mo17 × H95 and Mo17 × H95-*Rp1-D21*, respectively. The only substantial genetic difference between the components of each isogenic pair was at the *Rp1-D21* locus (Chaikam et al., 2011). A632 × H95*-Rp1-D21* used for the metabolic analysis and other lines used for SA assays were generated similarly. For the RNA sequencing (RNA-seq) analysis, plants were grown in the growth chamber in the North Carolina State University (NCSU) Phytotron at temperatures of 22 or 30°C in a 12 h light/12 h dark cycle. The experiments have two temperature treatments: (1) Plants were grown at constant 22°C for 18 days, and the fourth leaves were collected for RNA-seq analysis. (2) Plants were grown at 30°C for 14 days; then the temperature was dropped to 22°C to induce HR, and the fourth leaves were collected at 3, 6, 24, and 48 h post the temperature shift (hpts). Each sample was composed of pooled fourth leaves from five randomly chosen plants with two biological replicates. For the metabolic analysis, the isogenic hybrid pairs, A632 × H95 and A632 × H 95*-Rp1-D21* were grown in the field (Qingdao, China) and the fifth leaves at the V7 stage were collected with three biological replicates. For SA assays, the isogenic hybrid pairs in different genetic backgrounds were grown in the growth chamber for 18 days at a constant 22°C, and the fourth leaves were collected for SA measurement with three biological replicates.

Wild type *Nicotiana benthamiana* was grown at 24°C with a cycle of 16 h light and 8 h dark.

### Semi-quantitative RT-PCR

All primers used in this study are listed in Supplementary Table 8. Semi-quantitative RT-PCR was used to measure the gene expression as reported in our previous study (Negeri et al., 2013). Total RNA was extracted from the maize leaf tissue using Trizol (Life Technologies, Carlsbad, CA, USA) according to the instructions of the manufacturer. For complementary DNA (cDNA) synthesis, 1 μg of total RNA was reverse-transcribed using M-MLV (Life Technologies Corporation, Carlsbad, USA) following standard protocols. The amplification conditions for PCR consisted of 32 cycles of 94°C for 30 s, 57°C for 30 s, and 72°C for 30 s, using 250 μM of each primer and 1–2 μL of the 5 × diluted cDNA per reaction.

### RNA-Seq Library Construction and Transcriptome Sequencing

Total RNA was extracted from the fourth leaves pooled from five individuals collected from *Rp1-D21* mt and corresponding WT in B73 and Mo17 backgrounds at two treatments. The procedures for RNA sequencing (RNA-seq) analysis were performed according to our previous studies (Olukolu et al., 2014; Wang et al., 2015b). The quality and quantity of RNA were monitored by a NanoDrop 2000c spectrophotometer (Thermo Scientifc, Wilmington, DE, USA) and agarose gel electrophoresis. The messenger RNA (mRNA) was isolated from the total RNA by Dynabeadsoligo (dT25) (Invitrogen Life Technologies, MA, USA). The RNA-seq libraries were constructed according to the TruSeq RNA Sample Prep v2 LS as per the instructions of the manufacturer (Illumina Inc., CA, USA). The normalized libraries with individual index were loaded onto IlluminaHiSeq 2000 platform (Illumina Inc., San Diego, CA, USA) for cluster generation and sequencing. The data from two biological replicates were obtained by single end and paired-end reads (100-bp), respectively.

### Bioinformatic and Statistical Analyses

The sequencing reads were aligned to the maize B73 reference genome (ZmB73_RefGen_v38, ftp://ftp.ensemblgenomes.org) using hierarchical indexing for spliced alignment of transcripts 2 (HISAT2) (Kim et al., 2015) by the default parameter settings. Fragments per kilobase of exon model per million mapped reads (FPKM) and Pearson's correlation test were used for the estimation of gene transcription levels and the correlation between different biological replicates, respectively. To avoid taking the log of a number <1, all FPKM values were increased by 1. Differentially expressed genes (DEGs) were identified using the software package, edgeR, and DESeq2 from the Bioconductorsuite (Robinson et al., 2010; Love et al., 2014). The common DEGs were identified by both the methods with a false discovery rate (FDR) or adjusted *p*-values (padj) < 0.05 and a | Log2 (fold-change) | ≥1. Functional enrichment analysis of DEGs was performed based on Gene ontology (GO) Consortium database (http://www.geneontology.org). Network of DEGs and differentially accumulated metabolites (DAMs) were constructed based on Kyoto Encyclopedia of Genes and Genomes (KEGG) pathways (Kanehisa et al., 2017). A regulation overview of DEGs was accomplished using the MapMantool (Usadel et al., 2009). The common DEGs from 48 hpts and at 22°C grown condition treatments were selected to infer the gene regulatory networks (GRNs) using PlantTFDB v5.0 (http://plantregmap.cbi.pku.edu.cn; Zhou et al., 2020). The functional assignment of predicted transcription factors was done based on the functional classification information from the MapMan Toolkit.

### Metabolome Analysis

The fifth leaves of maize seedlings at V7 stage were collected with three replications for widely targeted metabolites analysis by ultra-performance liquid chromatography-mass spectrometry/Mass spectrometry (UPLC-MS/MS) (Chen et al., 2013). As described previously (Li et al., 2018; Mo et al., 2019), ~100 mg of freeze-dried leaf powder was dissolved in 0.6 mL of 70% methanol at 4°C overnight. Supernatant after centrifugation at 10, 000 g for 10 min was filtrated with a 0.22 μm pore size membrane for UPLC-MS/MS analysis using an LC-ESI-MS/MS system (HPLC, Shim-pack UFLC SHIMADZU CBM30A system, https://www.shimadzu.com.cn/; MS, Applied Biosystems 4500 Q TRAP, http://www.appliedbiosystems.com.cn/). Metabolite quantification is acquired with triple quadrupole scans using multi-reaction monitoring (MRM) model. The DAMs were identified by orthogonal partial least squares-discriminant analysis (OPLS-DA) according to the criteria of |fold-change| ≥ 1.5 and the variable importance in project (VIP) ≥1.

### SA Measurement

Salicylic acid was extracted as described previously (Wang et al., 2011). Briefly, 200 mg of each sample with three replications was ground into powder and extracted with 1.5 mL of 90% methanol followed by extraction with 1.5 mL of 100% methanol. 500 ng of *o*-anisic acid (Sigma, 169978) was added in each sample as the internal control. For each sample, 40 units of β-glucosidase (Sigma, G-0395) were added to digest glucosyl-conjugated SA (total SA) for 1.5 h at 37°C, and then were treated with an equal volume of 10% trichloroacetic acid (TCA) and centrifuged at 10,000 g for 10 min. A Dionex AS50 HPLC instrument with an Acclaim 120 C18 reverse column (4.6 3 250 mm) was used to detect the SA contents.

### UGTs Sequence Alignment and Phylogenetic Analysis

For phylogenetic analysis, the protein sequences from UDP-glucuronosyltransferase (UGT) family were aligned using Clustal X v2.1. Based on this alignment, a neighbor-joining tree was constructed using MEGA 6.0 software with 1,000 bootstrap replicates (Tamura et al., 2007). The neighbor-joining and *p*-distance methods were used with the pairwise deletion option to deal with gaps in the amino acid sequences.

### Plasmid Construction

Rp1-D21:HA, GUS:EGFP, and CC_D21_:EGFP were generated previously (Wang et al., 2015b). The cDNA sequences of ZmUGTs were isolated from B73 line and cloned into pENTR directional TOPO cloning vector (D-TOPO, Invitrogen, MA, USA). After sequencing, they were constructed into pSITEII-N1-EGFP vector (Martin et al., 2009) by LR reactions.

### *Agrobacterium tumefaciens*-Mediated Transient Expression

The *Agrobacterium tumefaciens* strain, GV3101 (pMP90) transformed with binary vector constructs was grown at 28°C overnight in 10 mL of L-broth medium supplemented with appropriate antibiotics. The detailed procedures were performed according to our previous study (Wang et al., 2015b). Unless otherwise indicated, all the experiments were repeated three times with similar results.

### Ion Leakage Measurement

Ion leakage conductivity was measured with a conductivity meter (METTLER TOLEDO, Zurich, Switzerland) according to our previous studies (Wang and Balint-Kurti, 2016; Zhu et al., 2020).

### Yeast Two-Hybrid Assay

ZmUGTs and CC_D21_ were respectively cloned into the pGADT7 (AD) and pGBKT7 (BD) vectors (Clontech, Mountain View, CA, USA) through LR reactions. The different combinations of AD- and BD-derived constructs were co-transformed into the yeast strain, Y2HGold. The Y2H assay was performed according to the protocol provided by the protocol of the manufacturer (Clontech, Mountain View, CA, USA).

### Protein Analysis and Co-immunoprecipitation (Co-IP) Assay

For protein analysis as described in our previous study, Myc-, EGFP- and HA-tagged constructs were transiently co-expressed in *Nicotiana benthamiana* and three leaf disk (~1.2 cm in diameter) from different *N. benthamiana* plants were collected at 30 h post inoculation **(**hpi). Total input protein was extracted in 150 μl extraction buffer (Wang et al., 2015b). Proteins for Co-IP assay were extracted from 0.6 g of leaf tissue collected at 40 hpi in 2.4 ml extraction buffer (Wang et al., 2015a; Wang and Balint-Kurti, 2016). The Western blot assays were performed according to our previous studies (Wang and Balint-Kurti, 2016; Zhu et al., 2020).

## Data Availability Statement

The original contributions presented in the study are publicly available. This data can be found at: National Center for Biotechnology Information (NCBI) BioProject database under accession number PRJNA288794 (https://www.ncbi.nlm.nih.gov/bioproject/PRJNA288794).

## Author Contributions

G-FW conceived the original research plans and supervised and designed the experiments. CG, Y-GW, and G-FW performed the experiments. CG, Y-GW, SL, XZ, B-KH, PB-K, and G-FW analyzed the data. CG and G-FW wrote the manuscript. PB-K revised the manuscript. All authors contributed to the article and approved the submitted version.

## Funding

This research was supported by grants from the National Natural Science Foundation of China (31871944, 31571263, and 32072405), Qilu Scholarship from Shandong University of China (11200086963061), Agricultural Variety Improvement Project of Shandong Province (2017LZN034), Natural Science Foundation of the USA Plant Genome grants (0822495 and 1444503), and the China Postdoctoral Science Foundation (2019M652391).

## Conflict of Interest

The authors declare that the research was conducted in the absence of any commercial or financial relationships that could be construed as a potential conflict of interest.

## Publisher's Note

All claims expressed in this article are solely those of the authors and do not necessarily represent those of their affiliated organizations, or those of the publisher, the editors and the reviewers. Any product that may be evaluated in this article, or claim that may be made by its manufacturer, is not guaranteed or endorsed by the publisher.
