# Supplementary material for: Multi-Omics Analyses Reveal the Regulatory Network and the Function of ZmUGTs in Maize Defense Response
Source: Front Plant Sci. 2021 Sep 24;12:738261. doi: 10.3389/fpls.2021.738261 (PMC8497902; doi:10.3389/fpls.2021.738261)
Supplement: Supplementary file 1 [file Data_Sheet_1.PDF]

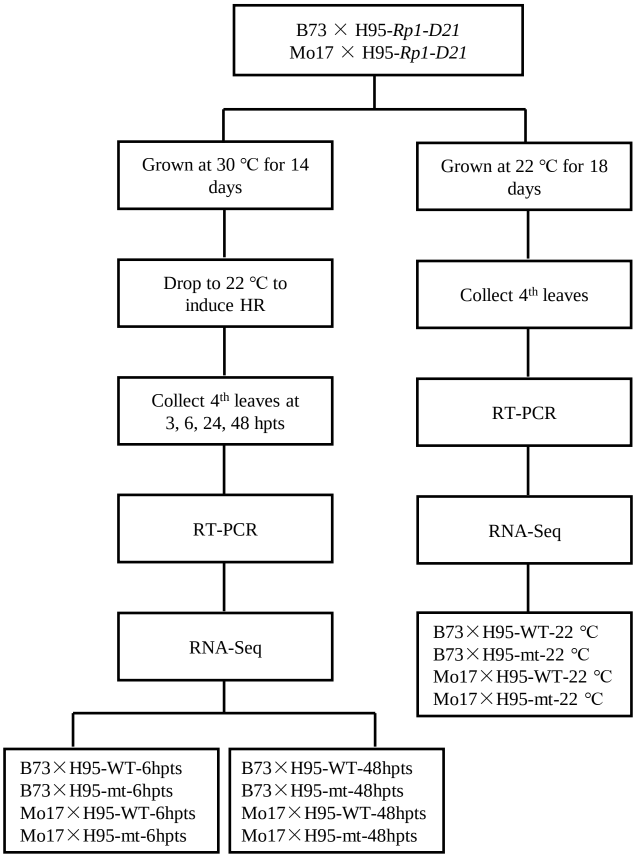

Supplementary Figure S1. Experimental design for RNA-seq analysis in wild type and *Rp1-D21* mutant collected from B73 × H95 and Mo17 × H95 backgrounds. The experiments have two temperature treatments: (1) Plants were grown at 30 ° C for 4 weeks (corresponding to the fourth leaf stage), then the temperature was dropped to 22 ° C to induce HR, and the 4th leaves were collected at 3, 6, 12, 24 and 48 hour post temperature shift (hpts). The samples at 6 and 48 hpts were used for RNA-seq analysis according to the transcript levels of *PR1* and *PR5*. (2) Plants were grown at constant 22 ° C for 6 weeks, and the 4th leaves were collected for RNA-seq analysis.

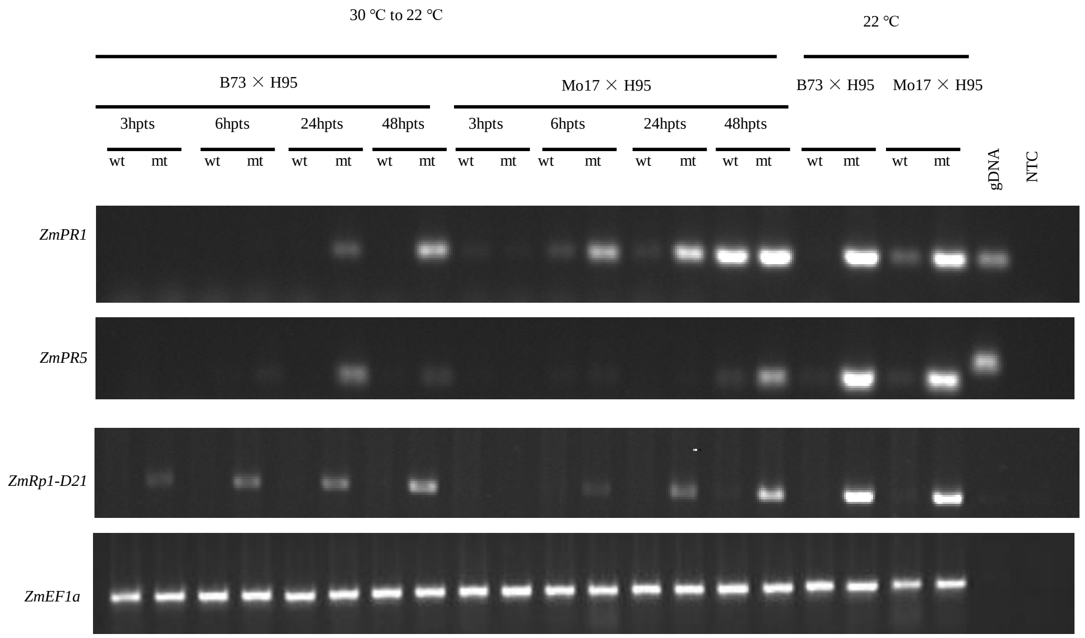

Supplemental Figure S2. The transcript levels of the defense marker genes *PR1*, *PR5* and *Rp1-D21* were detected by semi-quantitative PCR in wild type (WT) and *Rp1-D21* mutant (mt) collected from B73×H95 and Mo17×H95 backgrounds at 3 hours post temperature shift (hpts), 6 hpts, 24hpts and 48 hpts, and at constant 22 ℃, respectively.

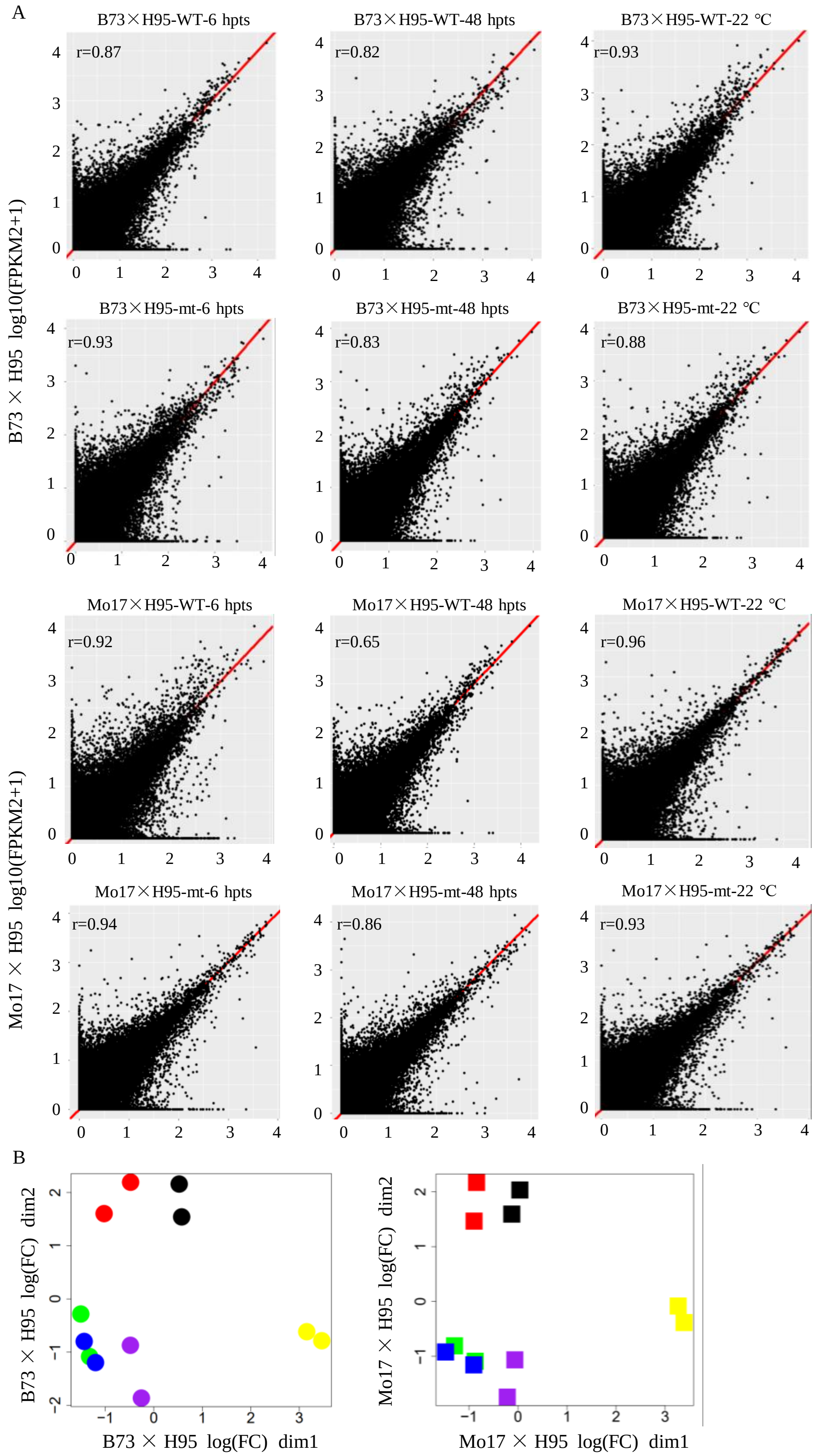

Supplemental Figure S3. Biological replicates validation of the RNA-seq data from different samples. (A) Correlation of two biological replicates in different treatments. Pair-wise Pearson's correlation (r) was calculated for different biological replicates. In each panel, the average log10 (FPKM1+1) value for each gene from one replicate is shown on the x-axis while the average log10 (FPKM2+1) value for each gene from the other replicate is shown on the y-axis. Wild type (WT) and *Rp1-D21* mutant (mt) were collected from B73×H95 and Mo17×H95 backgrounds. The seedlings were treated by two temperature conditions; one was that maize lines were grown in constant 22 ℃; the other was that plants were growth temperature at 30 ℃ for 14 days then dropped to 22 ℃ for 6 hpts and 48 hpts. hpts: hours post temperature shift. (B) The multi-dimensional scaling plot were performed to assess sequencing quality with all genes expressed in *Rp1-D21* mutant and its wild type at 6 hpts, 48 hpts and at constant 22 ° C. x- and y-axes represent the Euclidean distances between the samples. Green, blue, red, black, purple and yellow colors represent WT-6 hpts, mt-6 hpts, WT-48 hpts, mt-48 hpts, WT-22 ° C and mt-22 ° C in B73 ×H95 and Mo17 ×H95 backgrounds, respectively.

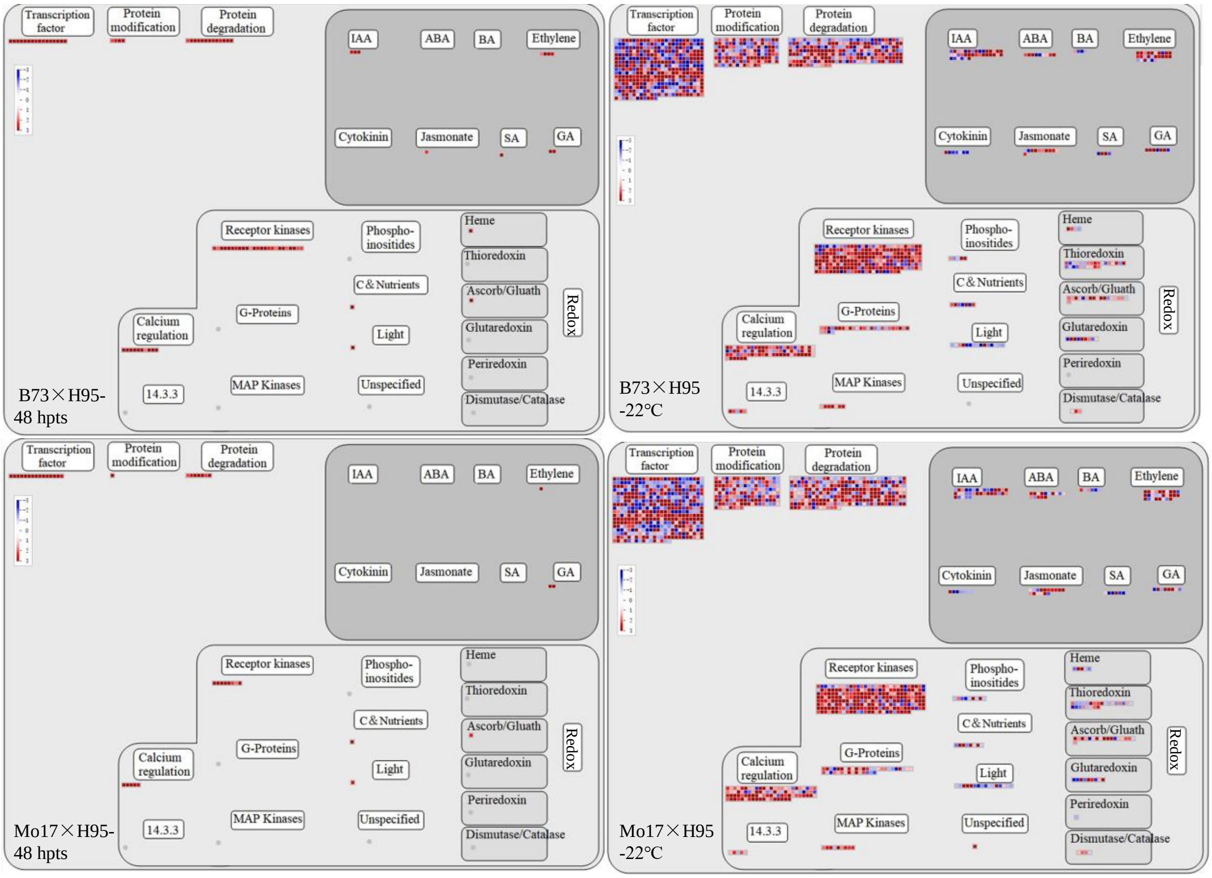

Supplemental Figure S4. Visualization of the DEGs in different pathways as analyzed by MapMan software. The DEGs were identified in *Rp1-D21* mutant compared to wild type at 48 hpts and at constant 22 ℃. Red and blue colors represent up-regulated genes and down-regulated genes, respectively.

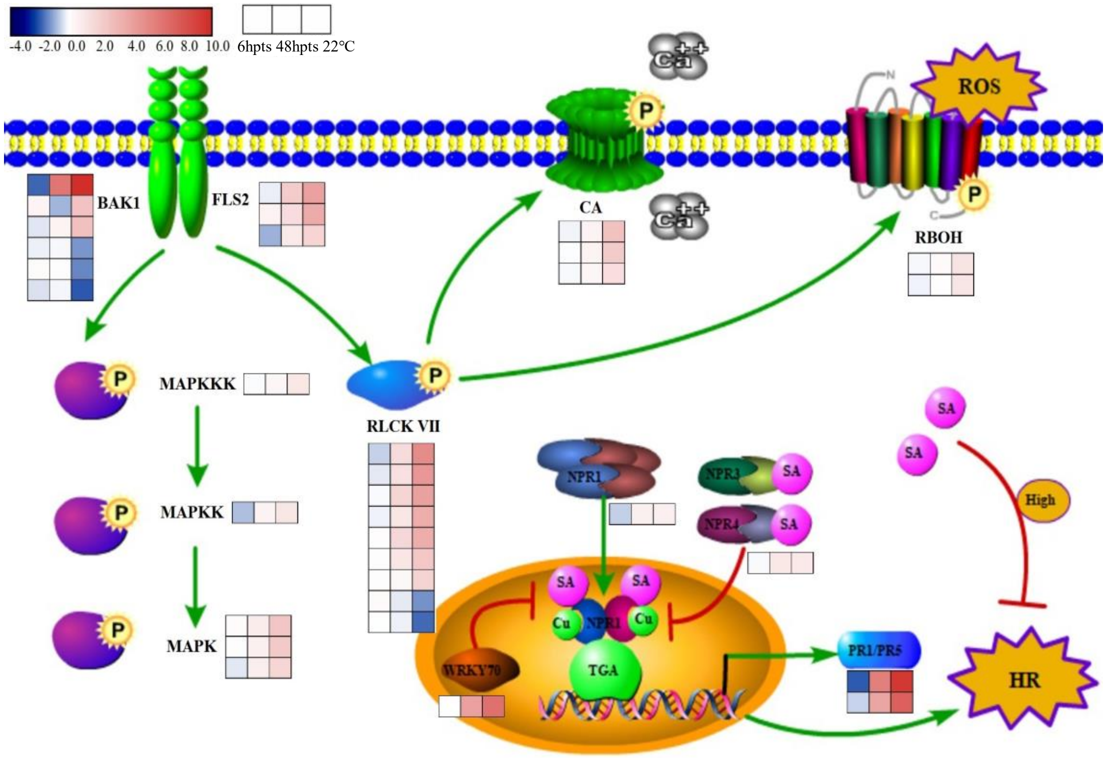

Supplemental Figure S5. Many genes in PTI are differentially expressed in *Rp1-D21* mutant compared to wild type. The heatmap represents the transcript levels of genes in PTI signaling pathway, and salicylic acid pathway at different time points. Red and blue colors represent up-regulated genes and down-regulated genes, respectively.

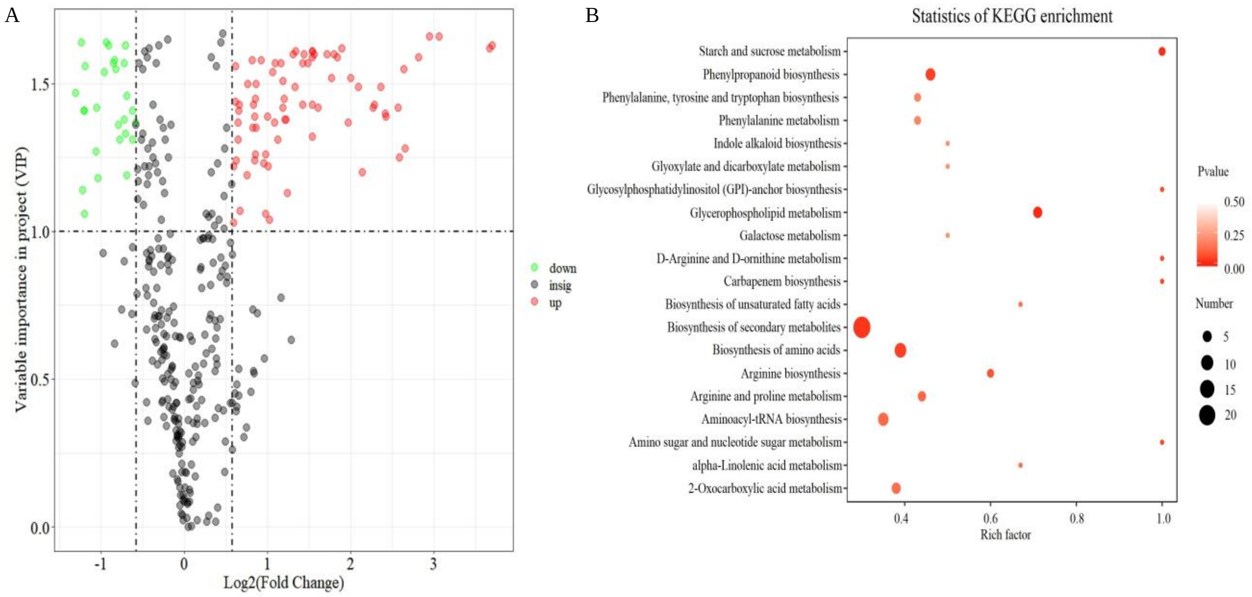

Supplemental Figure S6. The differential accumulated metabolites (DAMs) between the *Rp1-D21* mutant compared with wild type. A. The volcano distribution map of all DAMs. The X-axis represents the Fold Change in accumulation after conversion of the log2(Fold Change). The Y-axis represents the value of VIP. The green and red points represents the down- and up-regulated metabolites, respectively. B. The top twenty of KEGG enrichment of DAMs in *Rp1-D21* mutant compared to wild type. The size of the points in the graph represents the number of significantly differential metabolites enriched into the corresponding pathway.

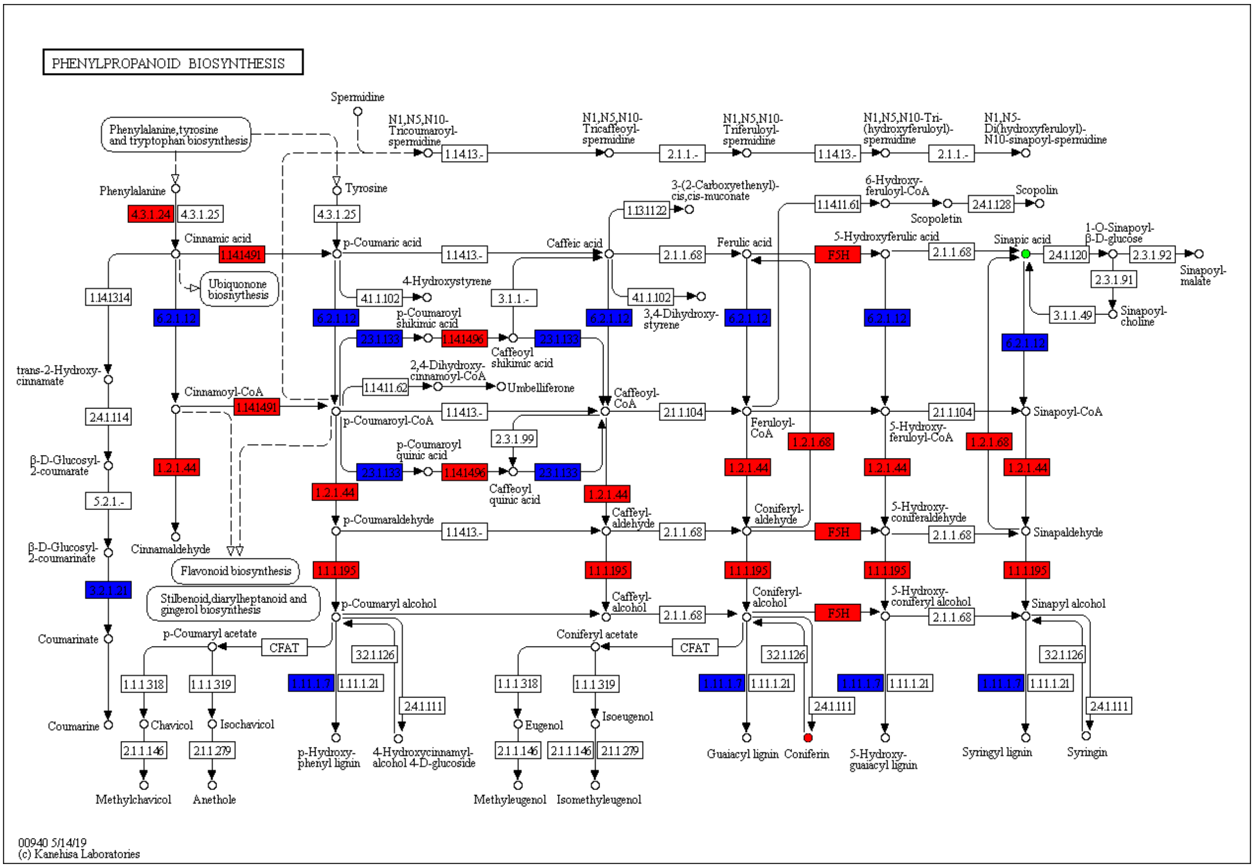

Supplemental Figure S7. The DEGs and DAMs enrichment in phenylpropanoid biosynthesis (Ko00940) in *Rp1-D21* mutant compared to wild type. Genes marked with red boxes indicate up-regulated DEGs, while genes marked with blue boxes contain both up- and down-regulated genes. Metabolites marked with red dot and green dot indicate up-regulated and down-regulated DAMs, respectively.

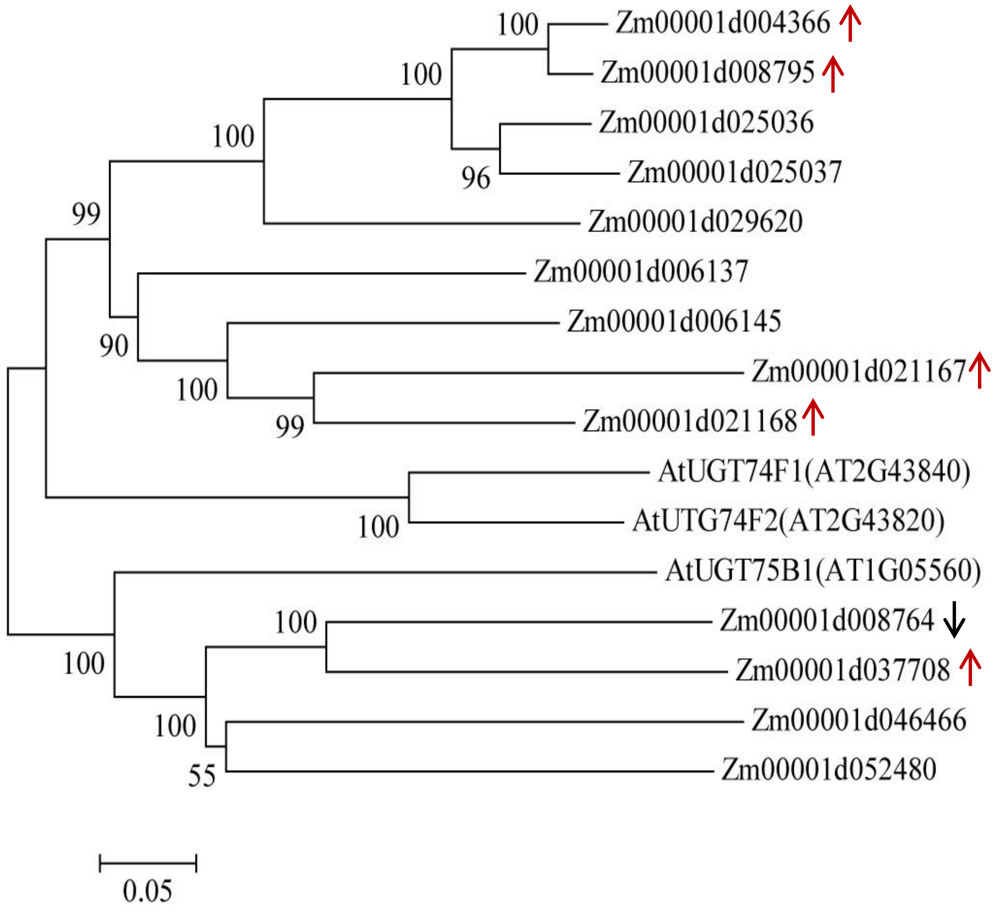

Supplemental Figure S8. Phylogenetic analysis of SAGT proteins. The protein sequences were aligned using Clustal X v2.1 and the phylogenetic tree was constructed using MEGA 6.0 software. At: *Arabidopsis thaliana*; Zm: *Zea mays*. The red and black arrows indicated the maize UGT homologs which were up- and down-expressed in *Rp1-D21* mutants compared to wild type.

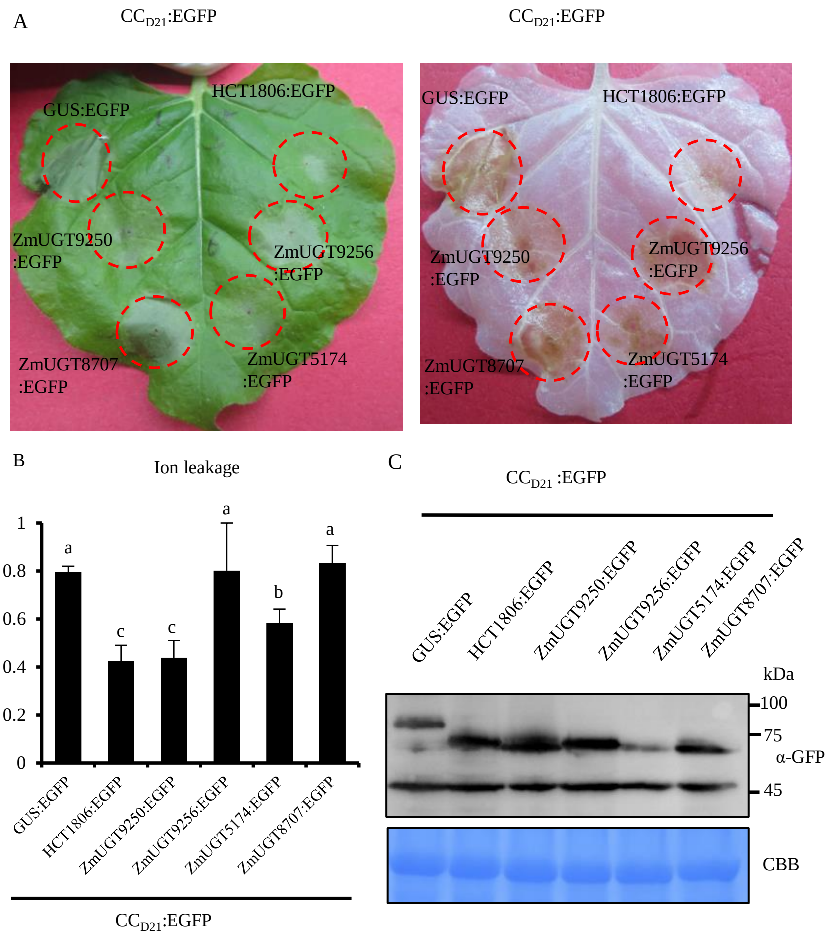

Supplemental Figure S9. Investigating the function of ZmUGTs in CC_{D21}-induced HR. A. ZmUGTs were transiently co-expressed with CC_{D21} into *N. benthamiana*. The representative leaf was photographed at 3 days after inoculation (left), and the same leaf was cleared by ethanol (right). B. Ion leakage conductivity (average ± standard error (SE), n > 5) was measured at 60 h after co-expression of GUS, HCT1806, ZmUGTs with CC_{D21}. Significant differences ($p < 0.05$) between samples are indicated by different letters (a–c). C. Total protein was extracted from agro-infiltrated leaves at 30 hpi. Anti-GFP was used to detect the expression of GUS, HCT1806, ZmUGTs and CC_{D21}. Equal loading of protein samples was shown by Coomassie brilliant blue (CBB) staining. The experiments were performed three times with similar results.

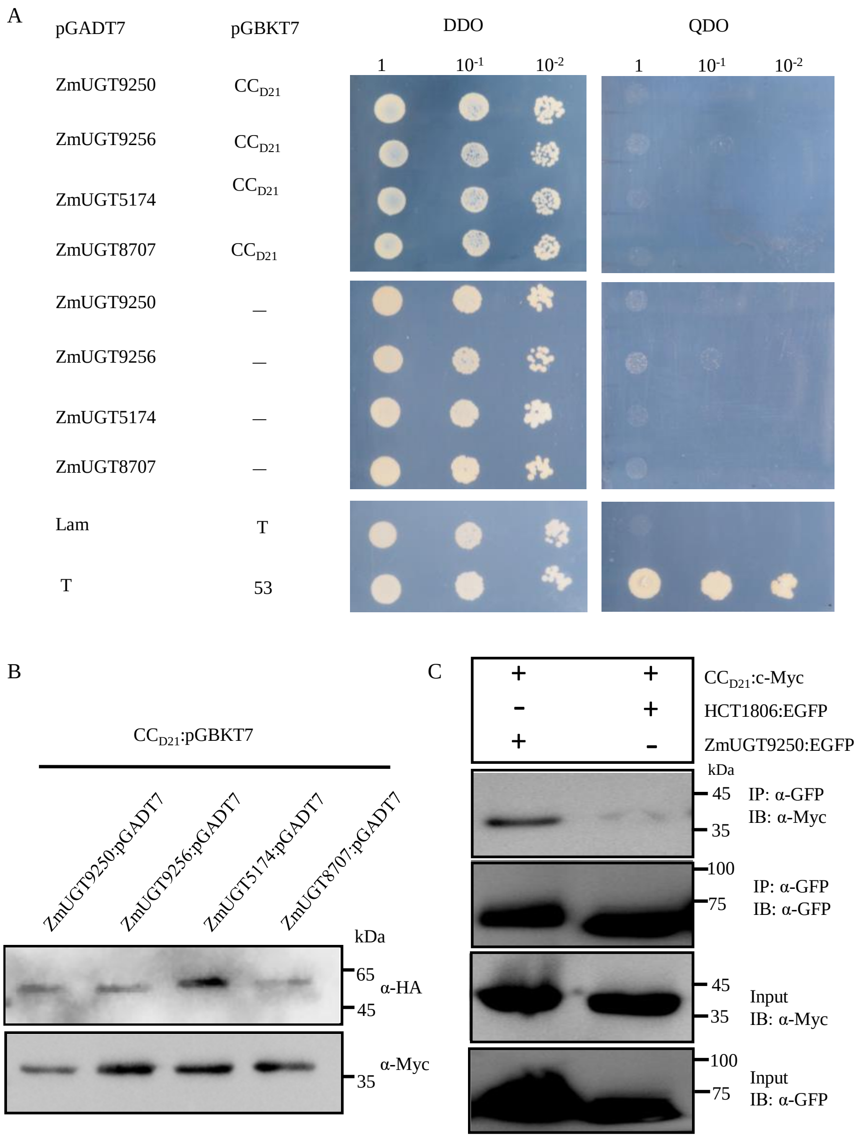

Supplemental Figure S10. Investigating the interactions between ZmUGTs and CC_{D21}. A. Investigating the interactions between ZmUGTs and CC_{D21} by yeast two hybridization (Y2H) assays. CC_{D21} were constructed into pGBKT7 and ZmUGTs were constructed into pGADT7. SV40 large T-antigen (T) with murine p53 (53) or Lam were used as the positive or negative controls. "–" indicated empty vector. DDO: SD-Leu-Trp; QDO: SD-Leu-Trp-Ade-His. B. Total protein was extracted from yeast co-expressed with different construct combinations from (A). Anti-HA and Anti-Myc were used to detect the expression of ZmUGTs and CC_{D21} respectively. C. Investigating the interactions between ZmUGT9250 and CC_{D21} by co-immunoprecipitation (Co-IP) assay. EGFP- and 4×c-Myc-tagged constructs were transiently co-expressed in *N. benthamiana* and samples were collected at 40 hpi for the Co-IP assay.

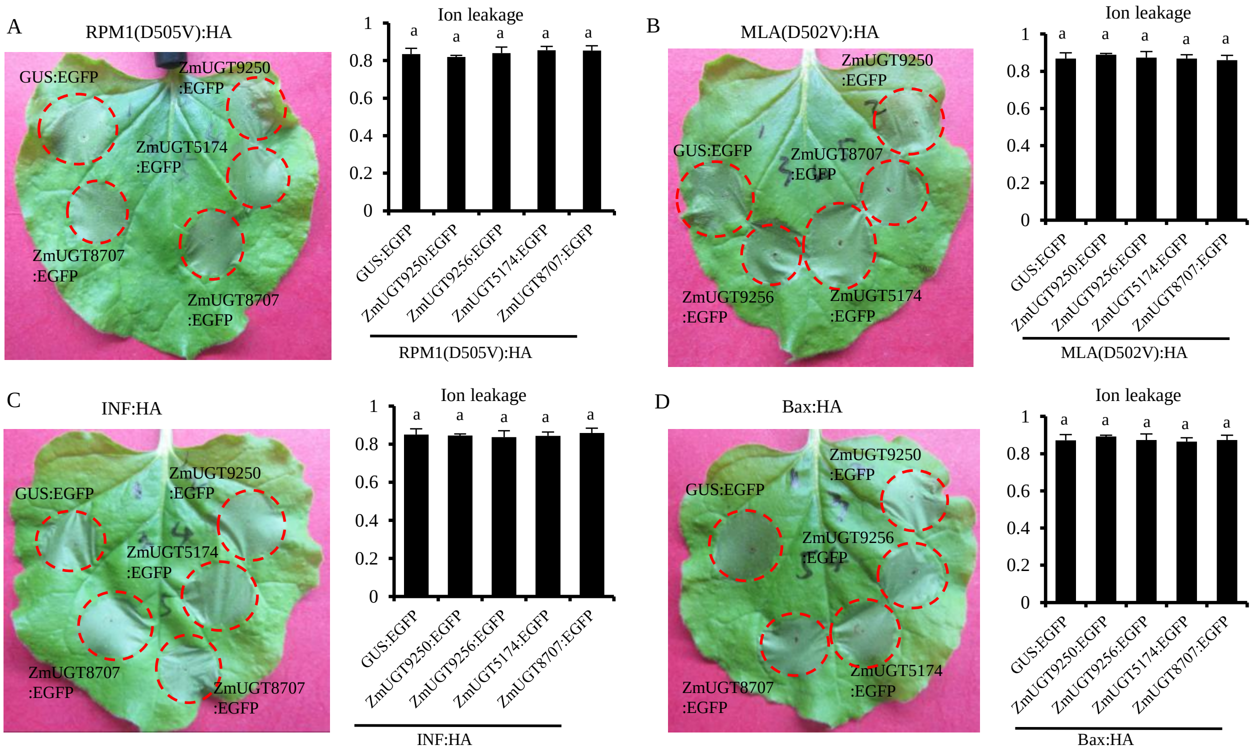

Supplemental Figure S11. Investigating the function of ZmUGTs in other elicitor-mediated cell death. ZmUGTs have no obvious suppressive roles on RPM1(D505V)-(A), MLA(D502V)-(B), INF1-(C) or Bax-(D) mediated cell death. GUS and ZmUGTs were transiently co-expressed with RPM1(D505V), MLA(D502V), INF1 or Bax into *N. benthamiana*. The representative leaves were photographed at 3 days after inoculation. Ion leakage conductivity (average ± standard error (SE), n > 5) was measured at 60 h after co-expression of GUS and ZmUGTs with different elicitors. Letter "a" means that there was no significant differences ($p < 0.05$) between samples. The experiments were performed three times with similar results.
